# Timing of vagus nerve stimulation during fear extinction determines efficacy in a rat model of PTSD

**DOI:** 10.1038/s41598-022-20301-9

**Published:** 2022-10-03

**Authors:** Rimenez R. Souza, Mark B. Powers, Robert L. Rennaker, Christa K. McIntyre, Seth A. Hays, Michael P. Kilgard

**Affiliations:** 1 Texas Biomedical Device Center, The University of Texas at Dallas, Richardson, TX 75080 USA; 2 School of Behavioral Brain Sciences, The University of Texas at Dallas, Bioengineering and Sciences Building, 14.506, 800 West Campbell Road, Richardson, TX 75080 USA; 3 Erik Jonsson School of Engineering and Computer Science, The University of Texas at Dallas, Richardson, TX 75080 USA; 4 Baylor University Medical Center, Dallas, TX 75246 USA

**Keywords:** Translational research, Psychology, Neuroscience, Emotion, Stress and resilience

## Abstract

Studies have indicated that vagus nerve stimulation (VNS) enhances extinction learning in rodent models. Here, we investigated if pairing VNS with the conditioned stimulus is required for the enhancing effects of VNS. Adult Sprague–Dawley rats were exposed to intense stress followed by fear conditioning training to produce resistant fear. Rats were then implanted with a cuff electrode around the left vagus. After recovery, rats underwent extinction training paired with VNS (0.5 s, 0.8 mA, 100 µs, and 30 Hz) or with Sham VNS (0 mA). VNS rats were randomized into the following subgroups: During VNS (delivered during presentations of the conditioned stimulus, CS), Between VNS (delivered between CS presentations), Continuous VNS (delivered during the entire extinction session), and Dispersed VNS (delivered at longer inter-stimulation intervals across the extinction session). Sham VNS rats failed to extinguish the conditioned fear response over 5 days of repeated exposure to the CS. Rats that received Between or Dispersed VNS showed modest improvement in conditioned fear at the retention test. During and Continuous VNS groups displayed the greatest reduction in conditioned fear. These findings indicate that delivering VNS paired precisely with CS presentations or continuously throughout extinction promotes the maximum enhancement in extinction learning.

## Introduction

Posttraumatic Stress Disorder (PTSD) is a highly debilitating psychiatric disorder that can develop in individuals exposed to a traumatic event^1,2^. The core of this disorder consists of exaggerated fear triggered by intrusive memories or other trauma reminders, avoidance of the trauma memories and other reminders, negative cognitions and mood, and hyperarousal/reactivity^3^. Exposure-based therapies are among the most empirically validated psychological therapies for PTSD^4–6^. These therapies aim to reframe the traumatic memories, creating new safe associations through extinction learning that in turn support the suppression of conditioned fear and decrease symptoms^7^. However, large clinical trials using exposure-based therapies for PTSD have yielded suboptimal response rates, incomplete symptom remission, and significant dropout^8–10^.

Impairments in the brain circuits that mediate extinction learning can underlie the development and persistence of PTSD^11–13^. Therefore, augmentation strategies that enhance extinction learning are expected to improve the benefits of exposure-based therapy^14,15^. Vagus nerve stimulation (VNS) is a promising strategy to augment the efficacy of exposure-based therapy. Preclinical studies in rodents have shown that VNS rapidly recruits the activity of neuromodulatory centers^16–18^, increasing the activity of norepinephrine, acetylcholine, and serotonin in brain areas critically involved in extinction learning such as the hippocampus, prefrontal cortex, and amygdala^19–22^. In addition, VNS engages the activity of several mediators of synaptic plasticity, structure, and neurogenesis, such as brain-derived neurotrophic factor (BDNF), fibroblast growth factor, NMDA receptor subunits, Arc protein, phosphorylated CaMKII and TrKB^23–27^. Studies in humans and rodents demonstrate that vagus nerve stimulation (VNS) enhances the consolidation of memories^28–30^, which suggests that this strategy could facilitate extinction learning^31,32^.

We have demonstrated in a number of recent studies that delivering VNS paired with the presentation of conditioned acoustic or olfactory cues during extinction training enhances extinction learning and reverses extinction impairments in rodent models for PTSD^22,25,33–37^. Importantly, studies indicate that choosing adequate stimulation parameters, including duration and intensity of stimulation, is critical in maximizing recovery after VNS-paired extinction^38,39^. In the same way that electrical stimulation parameters determine VNS efficacy^28,40–45^, the timing of VNS with respect to the concordant training regimens also critically determines efficacy^46–49^. Thus, the timing of VNS has the potential to influence its benefit to exposure-based therapy.

To probe the temporal determinants of VNS effects on extinction of conditioned fear, we tested the hypothesis that the extinction enhancement driven by VNS is dependent upon precisely pairing the conditioned stimulus to VNS bursts during extinction. To do so, we used a model for PTSD with severe stress and intense auditory fear conditioning that produces resistant extinction^50^. After this, rats underwent extinction training and received sham stimulation (0 mA) or 4 VNS bursts (0.8 mA, 8 s interval) delivered during or between each presentation of the conditioned stimulus. A subset of rats received the same number of VNS bursts dispersed throughout the extinction session ^32sinterval^. In addition, another subset of rats received VNS continuously throughout the sessions, at the same rate as in the VNS during group (8 s interval).

## Methods

### Animals

All methods followed the Animal Research: Reporting of In Vivo Experiments (ARRIVE) guidelines. The experiments were approved by the Institutional Animal Care and Use Committee (IACUC) at the University of Texas at Dallas (protocol number 15-13) and by the Animal Care and the Use Review Office (ACURO) of the United States Army Medical Research and Materiel Command Office of Research Protections, and conducted according to the protocols and guidelines. Eighty adult male Sprague–Dawley rats (Taconic Biosciences, USA), approximately 3 months old and weighing 250–280 g at the beginning of testing, were used in these studies. After arrival, rats were acclimated to the housing room for a week, and then were individually housed in plastic home cages. Three days before the beginning of the experiments, each rat was handled for 5 min a day in order to acclimate the animals to the experimenters. The animals were always kept under standard controlled room temperature, humidity, and light cycle (12:12 h, lights on at 6 am) and had free access to food and water. All procedures were conducted during the light cycle. Before the beginning of every procedure, a 20 min acclimation to the respective experimental room was used to reduce arousal.

### Rat model of PTSD

**Figure 1:**
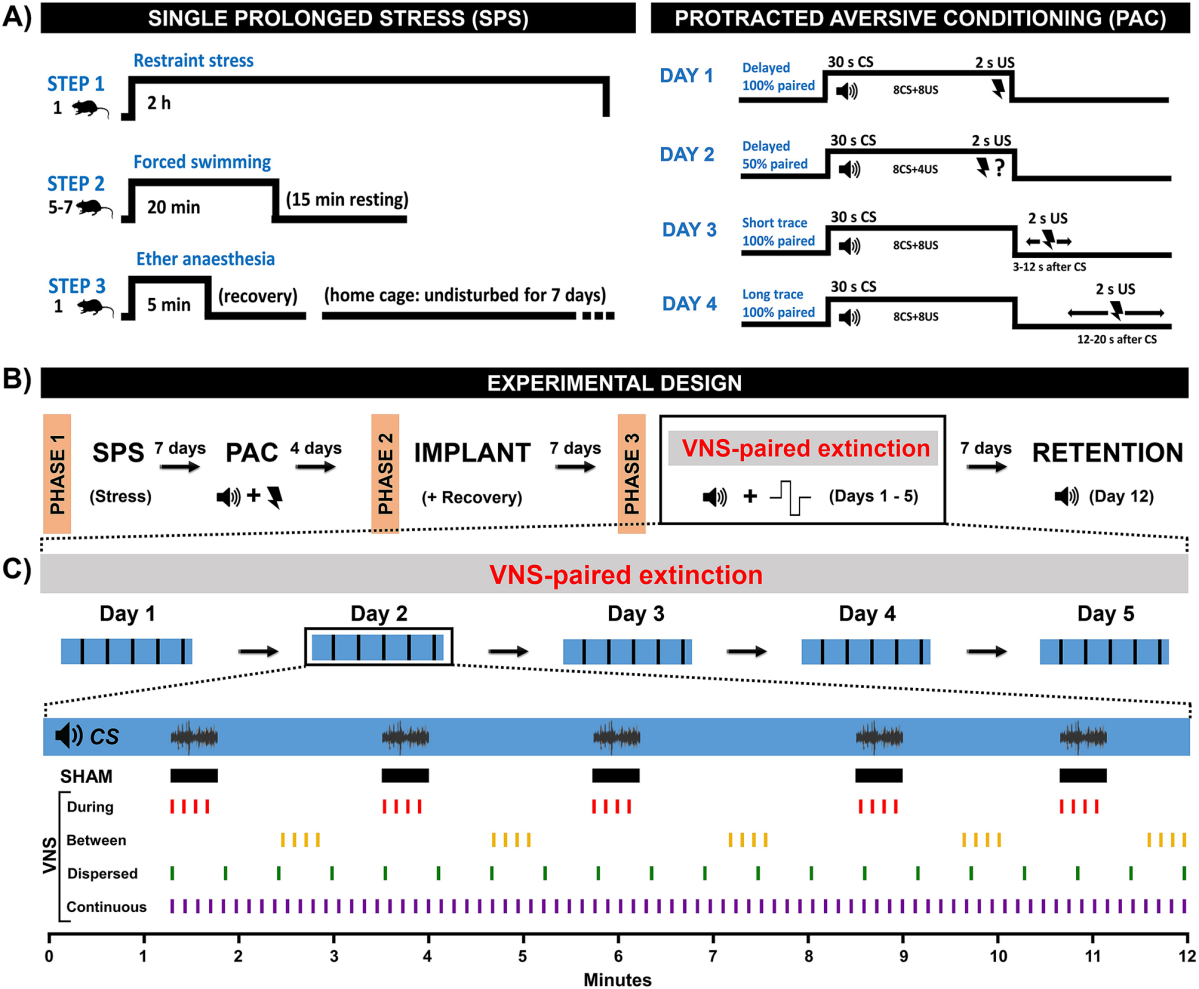
General methods, experimental design, and VNS parameters. (**A**) To assess the effects of different VNS intensities in extinction of severe fear, rats were submitted to the single prolonged stress (SPS) and protracted aversive conditioning (PAC). (**B**) In phase 1 of the experimental design, rats underwent SPS and remained undisturbed for 7 days. Fear conditioning was performed using the PAC over four days. In phase 2, rats were implanted with vagus nerve cuffs and allowed to recover for 6–7 days. In phase 3, rats were submitted to a five-day extinction training where Sham stimulation of VNS was delivered. Seven days later, all rats were tested for extinction retention in the same extinction context. (**C**) Rats in the *Sham* group received no stimulation. Rats in the VNS groups were randomly assigned to one of the following groups: during VNS (four 0.5-s bursts, at 8 s intervals and delivered with the onset of each CS trial); between VNS (four 0.5 s bursts, at 8 s interval and delivered between each CS trial); dispersed VNS (twenty 0.5 s stimulations, at 32 s interval distributed between the onset of the first trial and the end of the session); continuous VNS (eighty 0.5 s stimulations, at 8 s interval distributed between the onset of the first trial and the end of the session). All VNS rats received stimulations at 0.8 mA, 100 µs pulse width, at a frequency of 30 Hz. *SPS* single prolonged stress, *PAC* protracted aversive conditioning, *CS* conditioned stimulus, *US* unconditioned stimulus.

All rats were submitted to a model of PTSD that produces extinction-resistant fear (Fig. 1A), as we previously described^38^. In Phase 1, rats were submitted to the Single Prolonged Stress (SPS)^51^ procedure followed by the Protracted Aversive Conditioning (PAC) protocol seven days later^36,52^. The SPS model consisted of a 2 h of restraint stress, immediately followed by forced swimming in groups of 5–7 rats/tank during 20 min (water at 24 °C in 19 gal tank). After a 15 min recuperation period, rats were exposed to ether vapor until loss of responsiveness. Rats were then returned to clean home-cages and left undisturbed for 1 week.

One week after SPS, the PAC procedure was performed over four days using a fear conditioning box (22 × 20 × 20 cm) housed in a sound-attenuated chamber. The box was comprised of a grid floor connected to a shock generator, with a speaker mounted to the back wall. The shocker and the speaker were connected to a controller board and a computer triggered the stimuli using a custom MATLAB program. The conditioned stimulus (CS) was a complex sound of a warzone (guns firing, bombs, and aircraft engines), lasting 30 s and at an intensity ranging from 68 to 82 dB SPL. The broadband sound spanned a range of frequencies from 500 to 17,000 Hz. The variations in frequency bandwidth, frequency modulation, rise and decay times are described in our previous work^50^. A complex sound was selected over a pure tone because it more broadly activates the auditory cortex and requires a complex encoding process^53–55^, and it more closely resemble natural learning experiences. The unconditioned stimulus (US) was a scrambled footshock delivered through the grid floor at an intensity of 2.0 mA and duration of 2 s (HSCK100AP, Lafayette Instrument, USA). The inter-trial interval was pseudorandom, ranging from 90 to 180 s. At the end of each session, rats remained in the chamber for an additional two minutes before being transported to the home-cage.

On the first day, rats were twice exposed to the CS not followed by footshocks. After that, the CS was presented eight times such that they co-terminated with the US on every trial (delayed conditioning). On the second day, rats were exposed to eight CS’s that co-terminated with the US on 50% of the trials in a random order (unpredictable delayed conditioning). On the third day, rats were exposed to 8 CS’s paired with the US that was delivered randomly 3 to 12 s after the end of CS (short-trace conditioning). Finally, on the fourth day, rats were exposed to 8 CS’s paired with the US that was delivered randomly 12 to 20 s after the end of CS (long-trace conditioning). The apparatus was cleaned using 30% ethanol (v/v) after each rat was tested and with 70% ethanol (v/v) at the end of every day of testing.

### Cuff electrode and surgical procedures

In Phase 2, 4 days after the PAC procedure all rats were implanted with a VNS cuff, as we previously described^38^. This was performed to prevent any effect of the surgical procedures or anesthesia on the fear acquisition and to have an experimental design that parallels the clinical application of VNS surgery after the trauma (Fig. 1B). In brief, platinum-iridium wire electrodes were affixed to biocompatible micro-renathane cuffs (1.25 mm inner diameter, 2.5 mm outer diameter, 4.0 mm long). The two wires (7.5 cm long) were plugged to the micro strip connector (PS1-04-AA-LT, Omnetics, USA) used to connect the VNS cuff to a stimulator (Model 2100, A-M Systems, WA, USA). Right before implantation, cuff electrodes, bone screws and surgical tools were sterilized. The surgical procedure was performed under isoflurane anesthesia (2–3% in oxygen, Western Medical Supply, CA, USA) administered through inhalation. A 5 mm incision in the scalp and a 2 cm incision at the ventral midline above the tip of the sternum bone allowed the leads from the cuff to be tunneled subcutaneously to the top of the head. The sternomastoid, sternohyoid, and omohyoid muscles were blunt dissected and retracted, exposing the left vagus nerve adjacent to the carotid artery. After blunt dissection of the nerve from the carotid artery and other tissue, the cuff electrode was positioned around the nerve. Transient cessation of breathing was observed when stimulation (0.8 mA, 30 Hz, 5 s) was given through the implanted cuff, confirming that the electrode was appropriately positioned to stimulate the vagus nerve. The cuff was then tied using a silk suture, the muscle retractors were removed allowing the muscles to lie on their original position, and the incision was sutured. The rat was then attached to a stereotaxic frame, and a 15 mm incision was made on the scalp exposing the skull between the bregma and lambda sutures. A 4-pin connector was affixed to the skull using acrylic and bone screws, making a headcap mount. To prevent infection and reduce pain, each rat was given antibiotics (ceftriaxone, 4 mg/kg) and a nonsteroidal anti-inflammatory drug (ketoprofen, 5 mg/kg) subcutaneously. In addition, 10 ml of lactated Ringer’s in 5% dextrose was administered. Sham animals were subjected to the same surgical procedure, but were not stimulated during tests. All rats were allowed to recover for at least six days after surgery.

### VNS-paired extinction and retention test

After recovery from surgery, all rats underwent extinction training for five consecutive days, followed by an extinction retention test seven days later (Fig. 1B), as we previously described^38^. The extinction and the retention test were both conducted in Context B, an acrylic box housed in a sound-attenuated chamber. The apparatus was comprised of an irregular hexagon consisting of five clear acrylic walls (17 cm × 25 cm height) with honeycomb-shaped holes, and a clear solid front door (25 cm × 26 cm height), differing in shape, color, and flooring from where conditioning was performed. Both floor and ceiling were constructed of solid black acrylic. A commutator was installed above the ceiling and a small opening allowed a swivel to connect the commutator to the rat headcap. Stimulation was delivered by an isolated pulse stimulator (Model 2100, A-M Systems, USA) set with the following parameters: 0.5 s bursts, biphasic pulses of 0 (Sham) or 0.8 mA (active VNS) amplitude, at 100 µs pulse width, and frequency of 30 Hz^38,39^. A speaker of the same model as the one used in fear conditioning was mounted on the booth wall 10 cm from the back of the acrylic cage. The speaker and VNS commutator were connected to a controller board and a computer triggered the stimuli using a custom MATLAB program.

During extinction training rats received five CS presentations per day using the same ITI as in the conditioning. At the end of each session, rats remained in the chamber for an additional two minutes before being transported to the home-cage. To assess the temporal determinants of the efficacy of VNS to enhance extinction learning, the rats were randomly assigned to one of the following groups: Sham VNS (0 mA; n = 11), During VNS (n = 10), Between VNS (n = 11), Dispersed VNS (20 VNS bursts distributed between the first CS trial and the end of the session, at an inter-stimulation interval of 32 s; n = 9), and Continuous VNS (80 VNS bursts distributed between the first CS trial and the end of the session, at an inter-stimulation interval of 8 s; n = 11). For each cohort tested, small groups of 3–5 rats were randomized into treatments and allocated to balanced groups, ensuring that the groups were interleaved throughout the course of the experiment. Rats from all groups in each cohort were always tested simultaneously. Sham rats were always treated the same as VNS rats, except that no active stimulation was delivered during the extinction session. Apparatuses were cleaned between rats and at the end of every extinction session, as described for the conditioning procedure.

### Data analysis

Freezing was used as a measure of conditioned fear and was scored during each 30 s CS presentation. Freezing was defined as cessation of all movements other than the ones needed for respiration and sound location (brief and short head movements). All behavioral tests were scored by two experimenters who were blind to treatment conditions.

Data from extinction tests were analyzed using two-way repeated-measures ANOVA. Data from retention test were analyzed using one-way ANOVA. Bonferroni’s post hoc tests were performed to correct for multiple comparisons. Data from improvement figure were analyzed using paired t-tests. The minimum level of significance adopted was *p* < 0.05. Statistical analysis and graphical representation were performed using GraphPad Prism. Six rats were excluded from the experiment during extinction due to high cuff impedance (During VNS = 1; Between VNS = 2; Dispersed VNS = 2; Continuous VNS = 1). In addition, two rats were euthanized prior to extinction due to a failure of the headmount.

## Results

To evaluate the effects of VNS timing during extinction learning, we used a model for PTSD that we previously described to produce robust conditioned fear that is resistant to extinction^36,50^. Specifically, rats underwent an intense trauma consisting of the exposure to the SPS and PAC. Four days after PAC, rats underwent the implantation of a bipolar cuff electrode on the left cervical vagus nerve (Fig. 1A,B). After recovery, the rats received five days of extinction training paired with Sham VNS or one of four distinct VNS timing procedures: during VNS, between VNS, dispersed VNS, or continuous VNS (Fig. 1C).

**Figure 2:**
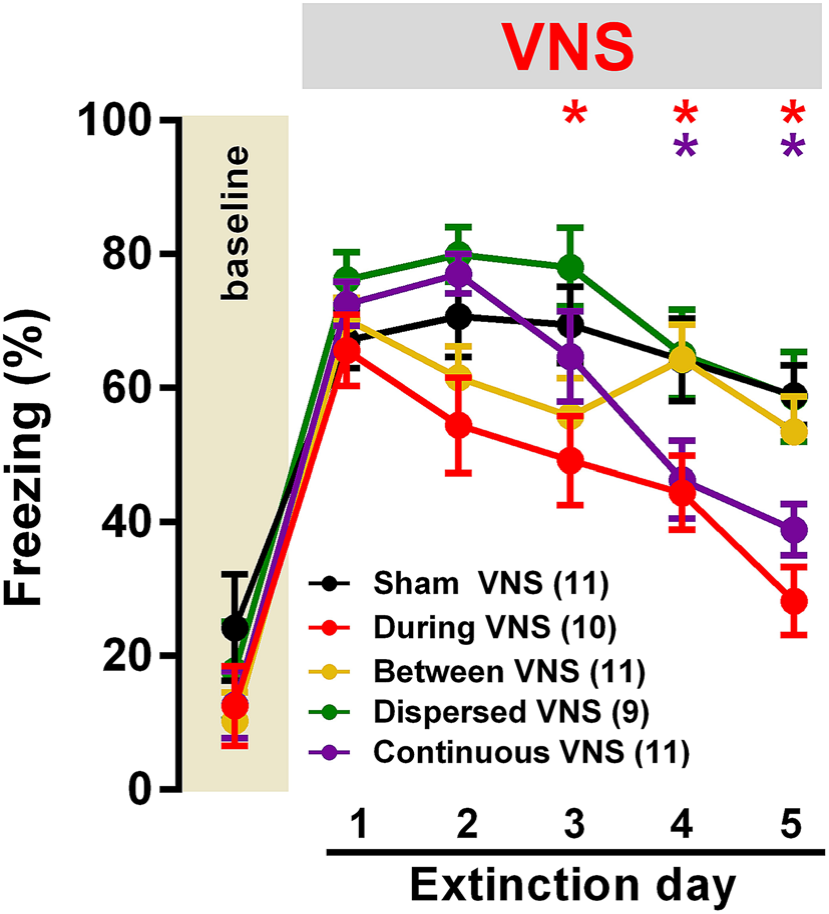
VNS reverses the impairment in extinction learning after severe stress and promotes fear reduction. Sham VNS rats failed to extinguish conditioned fear over 5 days of extinction training. Rats that received between VNS or dispersed VNS did not display an enhancement in extinction compared to Shams VNS rats. Significant reduction in conditioned fear in rats that received during VNS or continuous VNS indicates that the enhancing effects of VNS derive from concomitant presentation of the CS and stimulation of the vagus nerve. Top symbols indicate significant difference in comparison to Sham VNS rats on each respective day. Data are represented by the mean ± SEM using measures from each trial within sessions. Data was analyzed using two-way repeated-measures ANOVA. * *p* < 0.05 (Bonferroni’s post hoc test). Sham VNS (n = 11), during VNS (n = 10), between VNS (n = 11), dispersed VNS (n = 9), continuous VNS (n = 11).

Figure 2 shows the effects of VNS timing on extinction learning. No differences were found for baseline fear before CS presentation on day 1. A two-way repeated-measures ANOVA indicated a significant effect of treatment [F (4, 47) = 4.371, *p* < 0.005], day [F (5, 235) = 103.6, *p* < 0.0005], and an interaction [F (20, 235) = 2.274, *p* < 0.005] during the extinction phase (Fig. 2).

Bonferroni corrected post hoc tests revealed that the groups were not different at baseline before the first CS presentation on day 1 (*p* > 0.9). Compared to baseline, all groups showed significantly higher freezing during CS presentations on the first day of extinction (all *p*’s < 0.0001). This confirms that all groups acquired a strong conditioned response. Sham VNS rats did not display a reduction in conditioned responses across extinction days 1 to 5 (all *p*’s > 0.9). This confirms that SPS and PAC produce a conditioned fear response that is resistant to extinction.

Previous studies demonstrated that VNS delivered during the presentation of conditioned stimuli can boost extinction learning^22,34,35,38,39,56^. In this study, rats from the During VNS group similarly displayed a significant reduction in conditioned fear compared to Sham VNS on days 3 to 5 (all *p*’s < 0.05; Fig. 2). In addition, the During VNS group exhibited a significant reduction in conditioned fear on days 4 and 5 compared to day 1 of extinction (all *p*’s < 0.05; Fig. 2). These findings replicate earlier studies and confirm that VNS paired with CS trials reverses the extinction impairments after traumatic experiences that lead to maladaptive associative fear.

We next sought to determine if a matched amount of VNS delivered in between CS presentations could similarly enhance extinction (Fig. 1C). Despite receiving the same number of VNS bursts at the same interval, the Between VNS group did not show a significant reduction in conditioned fear compared to Sham VNS on any of the extinction days (all *p*’s > 0.2; Fig. 2). In addition, Between VNS failed to enhance extinction from day 1 to day 5 (*p* = 0.03), indicating that precisely timing VNS bursts might be critical to improve extinction learning.

Given that achieving precise VNS timing in the context of human exposure therapy can be difficult due to the complex nature of imaginal or in vivo exposures, we tested whether delivering VNS bursts throughout extinction training could reduce conditioned fear. A subset of rats received a matched amount of VNS bursts distributed across the session (Dispersed VNS), starting with the onset of the first CS trial at each session (Fig. 1C). Despite equivalent amount of VNS bursts in comparison to During VNS group, Dispersed VNS failed to boost extinction compared to Sham VNS rats or from days 1 to 5 (all *p*’s > 0.9; Fig. 2).

We next tested a simplified paradigm in which rats received a higher dose of VNS continuously across the session (continuous VNS) such that a matched number of VNS pairings occurring during CS presentation as received by rats in the During VNS (Fig. 1C). We found that the continuous VNS significantly reduced conditioned fear compared to the Sham VNS on days 4 and 5 (all *p*’s < 0.05). In addition, the Continuous VNS group showed a significant reduction in conditioned fear on days 4 and 5, compared to day 1 of extinction. The During and Continuous VNS groups exhibited significant reduction in conditioned fear compared to the Between VNS and Dispersed VNS groups (*p* < 0.05) at day 5, indicating that VNS timing determines efficacy. Together, these findings demonstrate that VNS can be delivered continuously at an effective rate to overcome the challenges in temporal dynamics of extinction learning and boost recovery.

**Figure 3:**
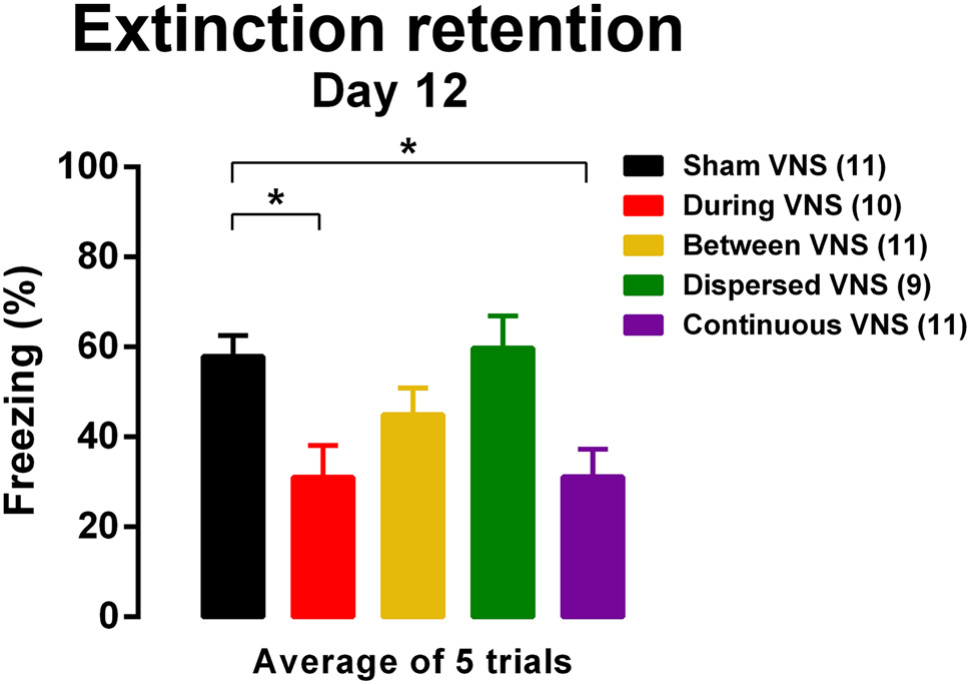
Extinction learning is maintained after 7 days of VNS-paired extinction. Rats that received during VNS or continuous VNS showed nearly 50% improvement in extinction recall compared to Sham VNS rats a week after extinction training. Data are represented by the mean ± SEM using the average of the 5 trials from the retention session, and analyzed by on-way ANOVA. *p < 0.05 (Bonferroni’s post hoc test). Sham (n = 11), VNS during (n = 10), VNS between (n = 11), VNS dispersed (n = 9), VNS continuous (n = 11).

We next tested the retention of extinction learning after extinction training. Seven days after the Sham VNS or VNS-paired extinction, the rats were re-exposed to the extinction context and received five presentations of the CS in the absence of any stimulation (Fig. 3). A one-way ANOVA indicated a significant effect of treatment [F (4, 47) = 4.946, *p* < 0.005]. Similar to enhancement of extinction, paradigms that ensured sufficient VNS delivery that co-occurred with CS presentation yielded the greatest retention of extinction. Bonferroni’s corrected post hoc tests revealed that the group that received During VNS and the group that received Continuous VNS showed a significant reduction in conditioned fear compared to Sham VNS group (all *p*’s < 0.05). No significant reduction in conditioned fear was found in the groups that received Between VNS or Dispersed VNS in the extinction phase. These results suggest that delivering VNS with CS presentations is required to support lasting extinction retention and promote sustained gains.

**Figure 4:**
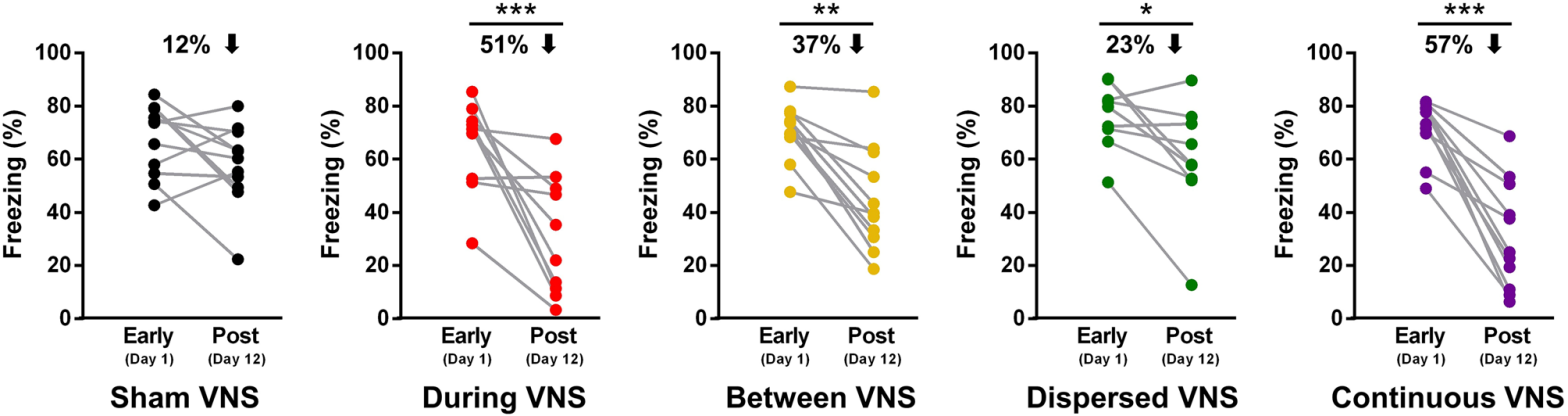
Percent of improvement from day 1 of extinction to retention test after extinction training. Sham VNS rats failed to show significant reduction in conditioned fear after extinction. All VNS groups showed significant reduction in conditioned fear, with the most robust effects seen in the groups that received During VNS or Continuous VNS. Data are represented by the mean ± SEM using the average of the five trials from each session, and analyzed by paired t-tests. *p < 0.05, **p < 0.005, ***p < 0.0005. Sham (n = 11), VNS during (n = 10), VNS between (n = 11), VNS dispersed (n = 9), VNS continuous (n = 11).

We also analyzed the gains in extinction of conditioned fear between the first day of extinction and the retention test, a week after extinction (Fig. 4). We found that Sham VNS rats were not able to extinguish the conditioned fear after 5 days of extinction training, and presented a negligible reduction in conditioned fear of 12% when retested a week after extinction [t(10) = 1.829, *p* = 0.09]. Interestingly, paired t-tests revealed that all VNS groups showed significant reductions in conditioned fear from the first day of extinction to the retention test. The group that received Dispersed VNS showed a 23% reduction in conditioned fear [t(8) = 2.876, *p* < 0.05], and the group that received Between VNS presented a reduction of 37% in conditioned fear [t(10) = 5.099, *p* < 0.005]. However, the two groups that received VNS paired with CS trials at the same rate, During VNS and Continuous VNS, displayed the greatest reductions in conditioned fear [During VNS = 52%; t(9) = 3.994, *p* < 0.005; Continuous VNS = 57%; (10) = 6.755, *p* < 0.0005]. Collectively, these findings indicate that VNS delivered concomitantly with CS trials produces robust enhancement in extinction that can resist spontaneous recovery of fear.

## Discussion

This study was designed to determine the role of VNS timing in the promotion of extinction learning in a rat model of PTSD. We provide an independent confirmation that VNS delivered during the presentations of a CS during extinction training can reverse extinction impairments^34,36,38,39^. The equivalent amount of VNS delivered between CS presentations or dispersed throughout the extinction session failed to decrease conditioned fear over five days of extinction training. It was possible to enhance extinction learning by delivering a greater amount of VNS that was continuously delivered throughout the entire extinction session. Thus, VNS enhances extinction learning when delivered during the presentation of fear cues. These findings may advance the translation of VNS therapy to improve the benefits of exposure-based therapy.

There is ample support for the use of exposure-based therapy in the treatment of PTSD. However, a substantial proportion of patients drop out of therapy prematurely or do not achieve complete symptom remission^8,57–61^. Therefore, there is a potential role for augmentation strategies that can improve the effectiveness of exposure-based therapy. VNS enhances memory consolidation in rodents and humans and has been proposed as a potential augmentation strategy for exposure-based therapy^28–30^. Studies in rodents indicate that VNS paired with extinction training enhances extinction and reverses extinction impairments in models of PTSD^22,25,34–36,39^. In addition, VNS-paired extinction produces extinction learning that can be resistant to different forms of fear relapse, such as spontaneous recovery, renewal, and reinstatement^34,36,38^. Further, pairing VNS with co-conditioned auditory or olfactory fear cues during fear extinction reduces fear to cues from the same or other modality that have not been extinguished^37,56^. Our findings that VNS paired with the conditioned stimulus presentation nearly doubled the reduction in conditioned fear at the end of the extinction phase provide an independent confirmation that VNS may be a promising strategy to augment exposure-based therapy^34,36,38^. In addition, the gains in fear remission seen at the end of the extinction phase were maintained a week after the extinction training, indicating that VNS can produce long-term symptom reduction. Collectively, these finds suggest that this strategy may improve recovery in the context of exposure-based therapy for PTSD.

Precisely pairing VNS with rehabilitative training might be critical to promote neural plasticity and improve recovery. Here we demonstrated that delivering VNS during, but not between CS presentations facilitated fear extinction, suggesting that temporal association between the memory reactivation and VNS is necessary for the enhancing effects of VNS. The observation that VNS must be delivered during fear cue presentation to be effective is consistent with previous studies in rodents showing that VNS paired with learning events facilitates the extinction of drug-seeking behavior and reversal learning^49,62^. These findings are in line with preclinical studies showing that VNS promotes neuronal plasticity and facilitates rehabilitation when delivered simultaneously with rehabilitation events^46–48,63^. This preclinical evidence is also supported by recent clinical studies demonstrating that precisely pairing VNS with sensory or rehabilitative training can improve recovery^64,65^.

Studies have shown that VNS enhances plasticity in the medial prefrontal cortex-basolateral amygdala pathway^22,62^, a key circuit that supports extinction learning^66^. This raises the possibility that the convergence of the extinction-evoked neuronal activity and the phasic increase in neuromodulatory function induced by VNS can strengthen neuronal processes that depend on rapid changes in synaptic excitability^67,68^. Earlier studies reported that the facilitating effects of infralimbic cortex stimulation on extinction learning are dependent upon a narrow temporal window around the conditioned stimulus onset^69–71^, which suggests that delivering VNS during fear memory recall might be key to enhancing extinction of conditioned cues.

Our observation that Continuous VNS (every 8 s) enhanced extinction learning while Dispersed VNS (every 32 s) did not enhance extinction confirms earlier observations that the dose of VNS also influences effectiveness^39,40,42,44,45,72,73^. This is supported by previous studies demonstrating that non-contingent VNS, but at a dose several times higher than that used in the Dispersed VNS condition in the present study, paired with drug-seeking behavior facilitated extinction and attenuated reinstatement^62^. It is important to note that VNS alone, in the absence of extinction training, does not reduce conditioned fear^35,37^ or promote plasticity in extinction circuits^22^. In addition, VNS delivered post-extinction or post-rehabilitative training for stroke does not enhance recovery^35,74^. Interestingly, the initial studies demonstrating that VNS enhances memory consolidation delivered post-training VNS to enhance the retention of an aversive memory in the inhibitory avoidance test^28,30^. Recent studies also demonstrated that post-training VNS can enhance the extinction of inhibitory avoidance^75^ and cocaine-induced conditioned place preference^76^. Such discrepancies in timing-dependent effects of VNS can possibly be attributed to the nature of the memory (*eg*. fear acquisition vs extinction) and the design of the task (*eg*. contextual vs cued learning), which likely influence the temporal dynamics and the circuits engaged during neuromodulatory activation elicited by VNS.

While the results of the present study are encouraging, some limitations deserve comment. First, PTSD affects nearly two times more women than men^1^, and the present study evaluated the effects of VNS in male rats only. While there are no known differences between males and females in previous studies assessing the effects of VNS paired with rehabilitative training^47,63,77–80^, marked sex differences have been described in circuits involved in fear learning and extinction^81,82^. Therefore, future studies should include both sexes and multiple assessments to unveil potential sex differences in the effects of VNS. Another limitation of the present study is that we did not assess the outcomes of VNS-paired extinction in long-term extinction retention. As fear extinction is not memory erasure, but rather new learning that suppresses maladaptive conditioned responses, fear may return spontaneously, in new contexts or after unrelated stressful experiences. Optimization of VNS parameters and therapy is expected to promote robust extinction and sustained gains^38,75^. Thus, future studies should determine whether VNS timing can influence long-term outcomes of extinction training. Finally, our study did not explore a neural signature that could explain why precisely pairing VNS with conditioned stimulus presentation is critical to enhance extinction. While the precise mechanisms that mediate VNS effects in extinction learning are largely unknown, it is likely that VNS produces time-dependent increases in synaptic excitability in brain circuits that support extinction learning^22^. Further studies using electrophysiological recordings and temporal control of extinction circuits during VNS-paired extinction are necessary to elucidate the mechanisms mediating the effects of VNS.

The present study builds on promising preclinical findings that VNS-paired extinction can enhance extinction learning, generalize the gains to conditioned stimuli not present during extinction^37,56^, attenuate fear return^38^, and reduce anxiety and other PTSD-like symptoms^34,56,83^. Collectively, these studies support the potential use of this strategy to maximize recovery using exposure-based therapy for PTSD.

### Translational considerations

The successful translation of VNS therapy from bench to bedside will be contingent on the identification of optimal methods to promote recovery^84,85^. Preclinical studies demonstrate that VNS delivered concurrent with the CS enhances the extinction of conditioned fear in rodents^22,33–37,56^, whereas stimulation delivered well after the presentation of the CS (present study) or after extinction training fails^35^. This complicates clinical implementation of VNS therapy for PTSD, which would entail delivering VNS during Prolonged Exposure (PE) therapy. Since exposure during PE is often imaginal, it is not possible to know in advance when the memory network will be engaged. In addition, imaginal exposure relies on individual ability to recall the traumatic experiences, and therapists rarely know with certainty which parts of a session of imaginal exposure will include a relevant CS. This would necessitate a therapist to identify the relevant moments during a session in order for VNS to be delivered and effectively provide augmentation.

**Figure 5:**
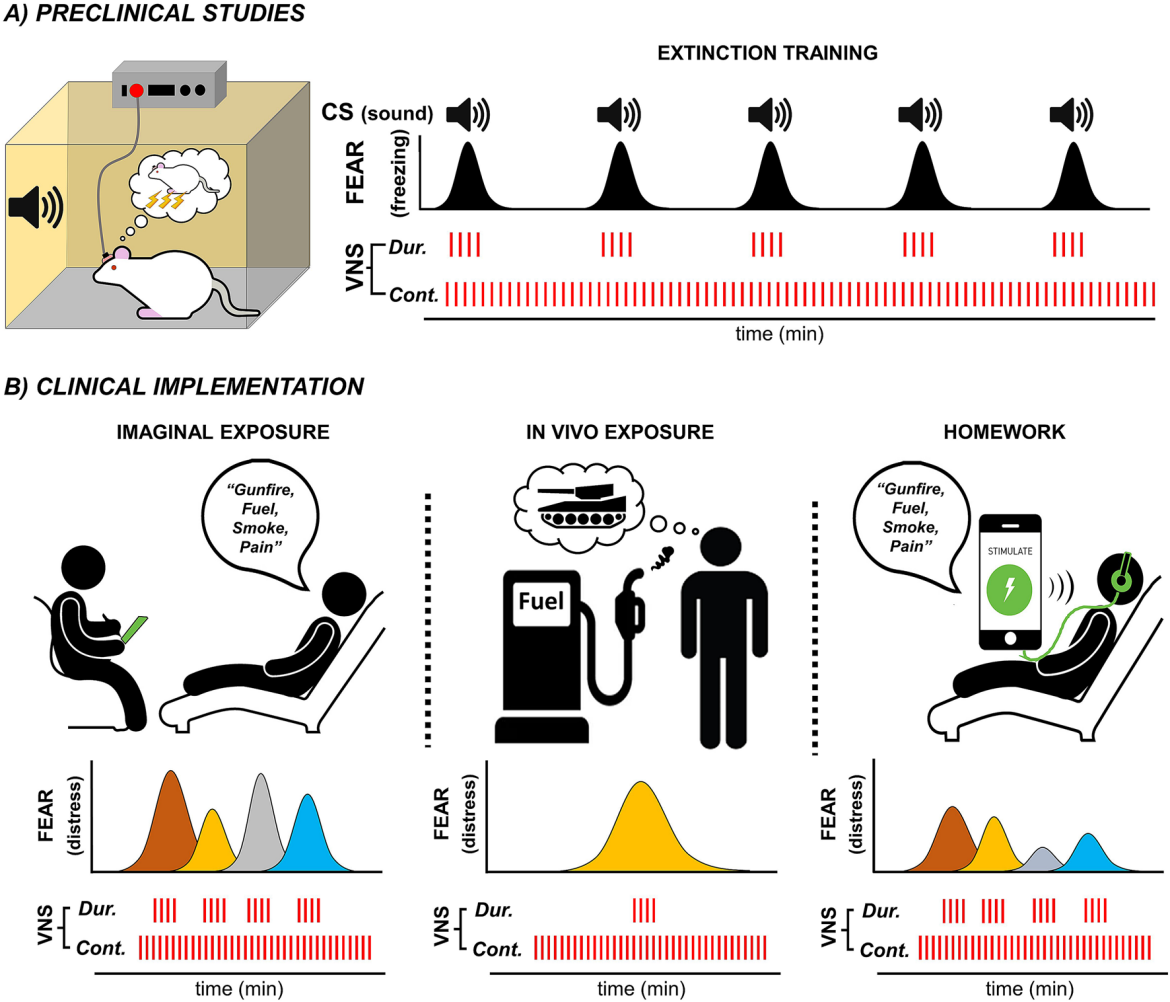
A schematic of the rationale for the translation of VNS into a clinical PTSD therapy based on preclinical observations. (**A**) Preclinical studies often use extinction of conditioned fear as a model of exposure therapy for PTSD. Fear (freezing) is evoked by presentations of the conditioned sound (CS) previously paired with a footshock. Delivering VNS during (Dur.) CS presentation only or continuously (Cont.) throughout the extinction session are equally effective at enhancing fear extinction in rats. (**B**) Since trauma cues are often complex, overlapping, and multimodal, clinical delivery of VNS during PTSD therapy is more complicated than it is in animal models. For example, therapists cannot readily control the intensity and duration of the recalled memories that drive distress. As a result, it may not be possible to deliver precisely paired VNS in exposure therapy as performed in preclinical studies in rodents. Our preclinical studies suggest that it may not be necessary to precisely time VNS delivery to occur only during peak recall of trauma reminders. We propose that VNS delivered continuously throughout imaginal exposure conducted by a therapist, in vivo exposure to trauma reminders, and homework assignments to listen to recorded therapy sessions may improve the effectiveness of prolonged exposure therapy.

In this study, we directly sought to define the necessity of CS-VNS pairing and to evaluate whether stimulation schemes that are easier to implement in a clinical setting would be effective at reducing fear. We confirmed that VNS delivered during each presentation of the CS that evokes conditioned fear in the rats successfully enhances fear extinction (Fig. 5A, “Dur.”). Importantly, we found that delivering continuous VNS during extinction equally facilitates extinction (Fig. 5A, “Cont.”). The CS presentations during fear extinction in this rat model mimic the trauma memory recall that produces distress in patients with PTSD undergoing PE, or other forms of exposure-based therapy. In PE, distress can be evoked while the patient is exploring details of the trauma with the therapist in the office (imaginal exposure), while exposing themselves to situations or stimuli that are perceived as dangerous or that remind them of the trauma (in vivo exposure), or during assigned activities such as listening to recordings of previous imaginal exposure (homework). Our findings suggest that therapists do not need to estimate or to find a behavioral marker to trigger VNS during the recall of details of a trauma (Fig. 5B, “Dur.”), and can simply stimulate throughout the session (Fig. 5B, “Cont.”). For example, the number, intensity, and duration of traumatic memory recalls during human imaginal exposure can vary substantially. Thus, continuous VNS stimulation during imaginal exposures can allow for an adequate VNS dose during therapy. The same is possible for in vivo exposures, in which the duration of the experience can be difficult to predict, or during homework, in which the continuous VNS therapy can be set to match a specific assignment, such as recorded imaginal exposures. This implementation of VNS therapy is currently being tested in people with PTSD (National Library of Medicine [NLM], NCT0406476290). The clinical trial utilizes the most efficient parameters identified in our pre-clinical studies, 0.5 s bursts of VNS every 8 s throughout imaginal, in vivo exposures and homework assignments during PE (Fig. 5B). This, and future clinical studies will reveal the potential of VNS therapy to augment the effects of PE for PTSD and determine whether adjustments in stimulation parameters and procedures are required in order to promote sustained gains after therapy.

## Supplementary Information

Supplementary Information.

## Data Availability

All datasets collected are included in the supplementary material.
