# Supplementary material for: Timing of vagus nerve stimulation during fear extinction determines efficacy in a rat model of PTSD
Source: Sci Rep. 2022 Oct 3;12:16526. doi: 10.1038/s41598-022-20301-9 (PMC9530175; doi:10.1038/s41598-022-20301-9)
Supplement: Supplementary file 1 — Supplementary Information. [file 41598_2022_20301_MOESM1_ESM.docx]

**Extinction day 1**

|  | Cohort | Rat | BL | CS1 | CS2 | CS3 | CS4 | CS5 | avg |
| --- | --- | --- | --- | --- | --- | --- | --- | --- | --- |
|  |  |  |  |  |  |  |  |  |  |
| SHAM | T1 | 5 | 30.0 | 76.7 | 78.3 | 75.0 | 75.0 | 73.3 | 75.7 |
| SHAM | T1 | 9 | 31.7 | 53.3 | 58.3 | 28.3 | 43.3 | 30.0 | 42.7 |
| SHAM | T1 | 14 | 16.7 | 80.0 | 81.7 | 68.3 | 81.7 | 85.0 | 79.3 |
| SHAM | T3 | 2 | 0.0 | 78.3 | 81.7 | 60.0 | 50.0 | 58.3 | 65.7 |
| SHAM | T3 | 8 | 56.7 | 53.3 | 58.3 | 50.0 | 55.0 | 36.7 | 50.7 |
| SHAM | T3 | 11 | 3.3 | 41.7 | 53.3 | 53.3 | 58.3 | 66.7 | 54.7 |
| SHAM | T3 | 14 | 6.7 | 75.0 | 40.0 | 68.3 | 61.7 | 45.0 | 58.0 |
| SHAM | T4 | 1 | 0.0 | 91.7 | 71.7 | 91.7 | 60.0 | 80.0 | 79.0 |
| SHAM | T4 | 6 | 43.3 | 83.3 | 95.0 | 76.7 | 81.7 | 85.0 | 84.3 |
| SHAM | T5 | 1 | 78.3 | 88.3 | 95.0 | 58.3 | 61.7 | 68.3 | 74.3 |
| SHAM | T5 | 7 | 0.0 | 68.3 | 68.3 | 80.0 | 73.3 | 78.3 | 73.7 |
| During | T1 | 1 | 43.3 | 78.3 | 78.3 | 80.0 | 80.0 | 78.3 | 79.0 |
| During | T1 | 3 | 0.0 | 75.0 | 78.3 | 78.3 | 61.7 | 63.3 | 71.3 |
| During | T1 | 11 | 0.0 | 60.0 | 26.7 | 15.0 | 18.3 | 21.7 | 28.3 |
| During | T1 | 13 | 0.0 | 38.3 | 53.3 | 51.7 | 55.0 | 58.3 | 51.3 |
| During | T1 | 16 | 50.0 | 86.7 | 86.7 | 78.3 | 86.7 | 88.3 | 85.3 |
| During | T3 | 4 | 11.7 | 80.0 | 76.7 | 71.7 | 68.3 | 75.0 | 74.3 |
| During | T3 | 6 | 8.3 | 56.7 | 81.7 | 61.7 | 93.3 | 71.7 | 73.0 |
| During | T3 | 13 | 11.7 | 63.3 | 51.7 | 56.7 | 40.0 | 51.7 | 52.7 |
| During | T4 | 15 | 0.0 | 66.7 | 81.7 | 43.3 | 85.0 | 71.7 | 69.7 |
| During | T5 | 5 | 0.0 | 80.0 | 83.3 | 85.0 | 70.0 | 38.3 | 71.3 |
| Between | T1 | 4 | 46.7 | 70.0 | 83.3 | 90.0 | 50.0 | 55.0 | 69.7 |
| Between | T1 | 6 | 13.3 | 55.0 | 65.0 | 63.3 | 81.7 | 76.7 | 68.3 |
| Between | T1 | 8 | 0.0 | 73.3 | 90.0 | 61.7 | 75.0 | 86.7 | 77.3 |
| Between | T1 | 12 | 1.7 | 71.7 | 81.7 | 83.3 | 75.0 | 78.3 | 78.0 |
| Between | T1 | 15 | 10.0 | 63.3 | 88.3 | 81.7 | 51.7 | 65.0 | 70.0 |
| Between | T3 | 3 | 5.0 | 65.0 | 70.0 | 36.7 | 33.3 | 33.3 | 47.7 |
| Between | T3 | 9 | 0.0 | 55.0 | 63.3 | 50.0 | 66.7 | 55.0 | 58.0 |
| Between | T3 | 15 | 10.0 | 73.3 | 83.3 | 71.7 | 76.7 | 66.7 | 74.3 |
| Between | T4 | 2 | 0.0 | 63.3 | 70.0 | 65.0 | 80.0 | 68.3 | 69.3 |
| Between | T4 | 11 | 3.3 | 90.0 | 90.0 | 88.3 | 75.0 | 93.3 | 87.3 |
| Between | T5 | 13 | 23.3 | 61.7 | 65.0 | 83.3 | 73.3 | 85.0 | 73.7 |
| Continuous | T3 | 5 | 3.3 | 75.0 | 65.0 | 36.7 | 48.3 | 50.0 | 55.0 |
| Continuous | T3 | 10 | 30.0 | 91.7 | 71.7 | 91.7 | 60.0 | 80.0 | 79.0 |
| Continuous | T3 | 12 | 13.3 | 56.7 | 83.3 | 60.0 | 85.0 | 81.7 | 73.3 |
| Continuous | T3 | 16 | 46.7 | 45.0 | 51.7 | 40.0 | 55.0 | 53.3 | 49.0 |
| Continuous | T4 | 4 | 3.3 | 58.3 | 73.3 | 76.7 | 71.7 | 78.3 | 71.7 |
| Continuous | T4 | 9 | 0.0 | 73.3 | 81.7 | 90.0 | 90.0 | 73.3 | 81.7 |
| Continuous | T4 | 12 | 0.0 | 88.3 | 83.3 | 91.7 | 75.0 | 66.7 | 81.0 |
| Continuous | T4 | 16 | 35.0 | 75.0 | 78.3 | 85.0 | 78.3 | 80.0 | 79.3 |
| Continuous | T5 | 8 | 5.0 | 86.7 | 88.3 | 83.3 | 60.0 | 86.7 | 81.0 |
| Continuous | T5 | 11 | 3.3 | 81.7 | 60.0 | 83.3 | 80.0 | 83.3 | 77.7 |
| Continuous | T5 | 16 | 0.0 | 66.7 | 81.7 | 43.3 | 85.0 | 71.7 | 69.7 |
| Dispersed | T4 | 5 | 0.0 | 85.0 | 75.0 | 65.0 | 65.0 | 71.7 | 72.3 |
| Dispersed | T4 | 10 | 60.0 | 91.7 | 93.3 | 93.3 | 88.3 | 85.0 | 90.3 |
| Dispersed | T4 | 14 | 6.7 | 68.3 | 80.0 | 58.3 | 78.3 | 71.7 | 71.3 |
| Dispersed | T5 | 2 | 23.3 | 60.0 | 95.0 | 90.0 | 58.3 | 30.0 | 66.7 |
| Dispersed | T5 | 4 | 5.0 | 73.3 | 58.3 | 50.0 | 31.7 | 43.3 | 51.3 |
| Dispersed | T5 | 6 | 0.0 | 73.3 | 81.7 | 90.0 | 90.0 | 73.3 | 81.7 |
| Dispersed | T5 | 10 | 48.3 | 80.0 | 86.7 | 81.7 | 78.3 | 85.0 | 82.3 |
| Dispersed | T5 | 12 | 10.0 | 85.0 | 70.0 | 83.3 | 81.7 | 78.3 | 79.7 |
| Dispersed | T5 | 15 | 6.7 | 78.3 | 93.3 | 95.0 | 91.7 | 91.7 | 90.0 |
|  |  |  |  |  |  |  |  |  |  |
| Broken cuff | T1 | 2 | 60.0 | 53.3 | 81.7 | 43.3 | 78.3 | 48.3 | 61.0 |
| Broken cuff | T1 | 7 | 21.7 | 88.3 | 86.7 | 78.3 | 90.0 | 80.0 | 84.7 |
| Broken cuff | T1 | 10 | 48.3 | 51.7 | 36.7 | 58.3 | 60.0 | 40.0 | 49.3 |
| Broken cuff | T4 | 7 | 38.3 | 93.3 | 86.7 | 70.0 | 93.3 | 83.3 | 85.3 |
| Broken cuff | T4 | 8 | 86.7 | 70.0 | 83.3 | 81.7 | 78.3 | 81.7 | 79.0 |
| Broken cuff | T4 | 13 | 3.3 | 81.7 | 60.0 | 83.3 | 80.0 | 83.3 | 77.7 |
| Excluded | T3 | 1 | 8.3 | 78.3 | 55.0 | 48.3 | 51.7 | 60.0 | 58.7 |
| Excluded | T3 | 7 | 6.7 | 45.0 | 51.7 | 31.7 | 51.7 | 46.7 | 45.3 |

**Extinction day 2**

| #NAME? | Cohort | Rat | CS1 | CS2 | CS3 | CS4 | CS5 | AVG |
| --- | --- | --- | --- | --- | --- | --- | --- | --- |
|  |  |  |  |  |  |  |  |  |
| SHAM | T1 | 5 | 53.3 | 76.7 | 81.7 | 33.3 | 53.3 | 59.7 |
| SHAM | T1 | 9 | 31.7 | 11.7 | 13.3 | 33.3 | 0.0 | 18.0 |
| SHAM | T1 | 14 | 48.3 | 65.0 | 81.7 | 71.7 | 75.0 | 68.3 |
| SHAM | T3 | 2 | 93.3 | 61.7 | 78.3 | 85.0 | 56.7 | 75.0 |
| SHAM | T3 | 8 | 83.3 | 85.0 | 90.0 | 86.7 | 75.0 | 84.0 |
| SHAM | T3 | 11 | 51.7 | 56.7 | 81.7 | 63.3 | 60.0 | 62.7 |
| SHAM | T3 | 14 | 86.7 | 86.7 | 78.3 | 86.7 | 88.3 | 85.3 |
| SHAM | T4 | 1 | 65.0 | 68.3 | 71.7 | 81.7 | 80.0 | 73.3 |
| SHAM | T4 | 6 | 85.0 | 81.7 | 68.3 | 68.3 | 73.3 | 75.3 |
| SHAM | T5 | 1 | 81.7 | 88.3 | 88.3 | 96.7 | 88.3 | 88.7 |
| SHAM | T5 | 7 | 71.7 | 93.3 | 91.7 | 91.7 | 88.3 | 87.3 |
| During | T1 | 1 | 65.0 | 81.7 | 75.0 | 46.7 | 58.3 | 65.3 |
| During | T1 | 3 | 46.7 | 40.0 | 43.3 | 28.3 | 38.3 | 39.3 |
| During | T1 | 11 | 31.7 | 31.7 | 6.7 | 15.0 | 10.0 | 19.0 |
| During | T1 | 13 | 45.0 | 20.0 | 10.0 | 16.7 | 3.3 | 19.0 |
| During | T1 | 16 | 60.0 | 68.3 | 36.7 | 58.3 | 45.0 | 53.7 |
| During | T3 | 4 | 70.0 | 81.7 | 78.3 | 66.7 | 73.3 | 74.0 |
| During | T3 | 6 | 71.7 | 56.7 | 51.7 | 58.3 | 61.7 | 60.0 |
| During | T3 | 13 | 61.7 | 81.7 | 40.0 | 45.0 | 48.3 | 55.3 |
| During | T4 | 15 | 76.7 | 83.3 | 75.0 | 56.7 | 85.0 | 75.3 |
| During | T5 | 5 | 85.0 | 95.0 | 68.3 | 90.0 | 78.3 | 83.3 |
| Between | T1 | 4 | 58.3 | 71.7 | 60.0 | 26.7 | 38.3 | 51.0 |
| Between | T1 | 6 | 65.0 | 61.7 | 55.0 | 61.7 | 61.7 | 61.0 |
| Between | T1 | 8 | 75.0 | 81.7 | 75.0 | 71.7 | 70.0 | 74.7 |
| Between | T1 | 12 | 61.7 | 46.7 | 51.7 | 58.3 | 31.7 | 50.0 |
| Between | T1 | 15 | 35.0 | 20.0 | 41.7 | 41.7 | 5.0 | 28.7 |
| Between | T3 | 3 | 61.7 | 48.3 | 53.3 | 61.7 | 50.0 | 55.0 |
| Between | T3 | 9 | 70.0 | 66.7 | 81.7 | 25.0 | 41.7 | 57.0 |
| Between | T3 | 15 | 70.0 | 85.0 | 76.7 | 71.7 | 70.0 | 74.7 |
| Between | T4 | 2 | 61.7 | 71.7 | 66.7 | 66.7 | 61.7 | 65.7 |
| Between | T4 | 11 | 85.0 | 83.3 | 81.7 | 68.3 | 75.0 | 78.7 |
| Between | T5 | 13 | 60.0 | 80.0 | 91.7 | 86.7 | 83.3 | 80.3 |
| Continuous | T3 | 5 | 71.7 | 73.3 | 83.3 | 66.7 | 88.3 | 76.7 |
| Continuous | T3 | 10 | 58.3 | 71.7 | 88.3 | 76.7 | 70.0 | 73.0 |
| Continuous | T3 | 12 | 85.0 | 75.0 | 71.7 | 85.0 | 53.3 | 74.0 |
| Continuous | T3 | 16 | 88.3 | 75.0 | 78.3 | 60.0 | 10.0 | 62.3 |
| Continuous | T4 | 4 | 53.3 | 61.7 | 56.7 | 71.7 | 66.7 | 62.0 |
| Continuous | T4 | 9 | 63.3 | 78.3 | 83.3 | 73.3 | 63.3 | 72.3 |
| Continuous | T4 | 12 | 83.3 | 81.7 | 83.3 | 70.0 | 75.0 | 78.7 |
| Continuous | T4 | 16 | 81.7 | 83.3 | 85.0 | 88.3 | 86.7 | 85.0 |
| Continuous | T5 | 8 | 85.0 | 85.0 | 85.0 | 68.3 | 88.3 | 82.3 |
| Continuous | T5 | 11 | 95.0 | 86.7 | 86.7 | 88.3 | 90.0 | 89.3 |
| Continuous | T5 | 16 | 90.0 | 93.3 | 86.7 | 95.0 | 95.0 | 92.0 |
| Dispersed | T4 | 5 | 83.3 | 75.0 | 78.3 | 76.7 | 61.7 | 75.0 |
| Dispersed | T4 | 10 | 78.3 | 80.0 | 93.3 | 85.0 | 86.7 | 84.7 |
| Dispersed | T4 | 14 | 90.0 | 86.7 | 85.0 | 68.3 | 81.7 | 82.3 |
| Dispersed | T5 | 2 | 56.7 | 90.0 | 61.7 | 46.7 | 56.7 | 62.3 |
| Dispersed | T5 | 4 | 71.7 | 78.3 | 53.3 | 56.7 | 35.0 | 59.0 |
| Dispersed | T5 | 6 | 65.0 | 91.7 | 93.3 | 91.7 | 93.3 | 87.0 |
| Dispersed | T5 | 10 | 78.3 | 93.3 | 96.7 | 96.7 | 88.3 | 90.7 |
| Dispersed | T5 | 12 | 91.7 | 88.3 | 80.0 | 81.7 | 75.0 | 83.3 |
| Dispersed | T5 | 15 | 93.3 | 96.7 | 95.0 | 96.7 | 95.0 | 95.3 |
|  |  |  |  |  |  |  |  |  |
| Broken cuff | T1 | 2 | 70.0 | 65.0 | 76.7 | 48.3 | 33.3 | 58.7 |
| Broken cuff | T1 | 7 | 60.0 | 41.7 | 30.0 | 35.0 | 18.3 | 37.0 |
| Broken cuff | T1 | 10 | 71.7 | 58.3 | 56.7 | 53.3 | 70.0 | 62.0 |
| Broken cuff | T4 | 7 | 73.3 | 78.3 | 71.7 | 85.0 | 86.7 | 79.0 |
| Broken cuff | T4 | 8 | 78.3 | 71.7 | 80.0 | 81.7 | 68.3 | 76.0 |
| Broken cuff | T4 | 13 | 68.3 | 85.0 | 86.7 | 83.3 | 80.0 | 80.7 |
| Excluded | T3 | 1 | 0.0 | 0.0 | 0.0 | 0.0 | 0.0 | 0.0 |
| Excluded | T3 | 7 | 0.0 | 0.0 | 0.0 | 0.0 | 0.0 | 0.0 |

**Extinction day 3**

|  | Cohort | Rat | CS1 | CS2 | CS3 | CS4 | CS5 | AVG |
| --- | --- | --- | --- | --- | --- | --- | --- | --- |
|  |  |  |  |  |  |  |  |  |
| SHAM | T1 | 5 | 70.0 | 75.0 | 66.7 | 48.3 | 63.3 | 64.7 |
| SHAM | T1 | 9 | 38.3 | 40.0 | 11.7 | 21.7 | 13.3 | 25.0 |
| SHAM | T1 | 14 | 88.3 | 68.3 | 81.7 | 71.7 | 71.7 | 76.3 |
| SHAM | T3 | 2 | 66.7 | 85.0 | 65.0 | 61.7 | 66.7 | 69.0 |
| SHAM | T3 | 8 | 55.0 | 41.7 | 33.3 | 40.0 | 51.7 | 44.3 |
| SHAM | T3 | 11 | 75.0 | 76.7 | 76.7 | 88.3 | 83.3 | 80.0 |
| SHAM | T3 | 14 | 73.3 | 88.3 | 58.3 | 78.3 | 65.0 | 72.7 |
| SHAM | T4 | 1 | 81.7 | 81.7 | 60.0 | 76.7 | 85.0 | 77.0 |
| SHAM | T4 | 6 | 81.7 | 93.3 | 83.3 | 85.0 | 85.0 | 85.7 |
| SHAM | T5 | 1 | 68.3 | 83.3 | 93.3 | 85.0 | 88.3 | 83.7 |
| SHAM | T5 | 7 | 68.3 | 88.3 | 91.7 | 91.7 | 88.3 | 85.7 |
| During | T1 | 1 | 73.3 | 78.3 | 81.7 | 53.3 | 58.3 | 69.0 |
| During | T1 | 3 | 61.7 | 53.3 | 75.0 | 53.3 | 63.3 | 61.3 |
| During | T1 | 11 | 46.7 | 28.3 | 21.7 | 21.7 | 5.0 | 24.7 |
| During | T1 | 13 | 26.7 | 33.3 | 36.7 | 21.7 | 28.3 | 29.3 |
| During | T1 | 16 | 58.3 | 51.7 | 33.3 | 28.3 | 23.3 | 39.0 |
| During | T3 | 4 | 66.7 | 66.7 | 58.3 | 20.0 | 31.7 | 48.7 |
| During | T3 | 6 | 38.3 | 36.7 | 33.3 | 36.7 | 30.0 | 35.0 |
| During | T3 | 13 | 60.0 | 26.7 | 8.3 | 36.7 | 10.0 | 28.3 |
| During | T4 | 15 | 71.7 | 85.0 | 63.3 | 85.0 | 81.7 | 77.3 |
| During | T5 | 5 | 86.7 | 63.3 | 83.3 | 76.7 | 85.0 | 79.0 |
| Between | T1 | 4 | 38.3 | 51.7 | 30.0 | 25.0 | 33.3 | 35.7 |
| Between | T1 | 6 | 46.7 | 48.3 | 25.0 | 61.7 | 75.0 | 51.3 |
| Between | T1 | 8 | 65.0 | 71.7 | 71.7 | 61.7 | 80.0 | 70.0 |
| Between | T1 | 12 | 58.3 | 71.7 | 66.7 | 60.0 | 40.0 | 59.3 |
| Between | T1 | 15 | 60.0 | 40.0 | 43.3 | 51.7 | 45.0 | 48.0 |
| Between | T3 | 3 | 38.3 | 13.3 | 10.0 | 35.0 | 18.3 | 23.0 |
| Between | T3 | 9 | 43.3 | 55.0 | 28.3 | 35.0 | 48.3 | 42.0 |
| Between | T3 | 15 | 63.3 | 50.0 | 58.3 | 31.7 | 63.3 | 53.3 |
| Between | T4 | 2 | 66.7 | 75.0 | 76.7 | 83.3 | 76.7 | 75.7 |
| Between | T4 | 11 | 90.0 | 91.7 | 86.7 | 83.3 | 93.3 | 89.0 |
| Between | T5 | 13 | 78.3 | 90.0 | 65.0 | 85.0 | 13.3 | 66.3 |
| Continuous | T3 | 5 | 70.0 | 56.7 | 36.7 | 36.7 | 56.7 | 51.3 |
| Continuous | T3 | 10 | 53.3 | 71.7 | 80.0 | 33.3 | 58.3 | 59.3 |
| Continuous | T3 | 12 | 76.7 | 75.0 | 1.7 | 5.0 | 3.3 | 32.3 |
| Continuous | T3 | 16 | 23.3 | 31.7 | 21.7 | 23.3 | 21.7 | 24.3 |
| Continuous | T4 | 4 | 80.0 | 88.3 | 85.0 | 80.0 | 85.0 | 83.7 |
| Continuous | T4 | 9 | 78.3 | 88.3 | 85.0 | 83.3 | 75.0 | 82.0 |
| Continuous | T4 | 12 | 90.0 | 80.0 | 90.0 | 81.7 | 71.7 | 82.7 |
| Continuous | T4 | 16 | 86.7 | 26.7 | 86.7 | 20.0 | 26.7 | 49.3 |
| Continuous | T5 | 8 | 88.3 | 85.0 | 76.7 | 68.3 | 85.0 | 80.7 |
| Continuous | T5 | 11 | 86.7 | 90.0 | 88.3 | 55.0 | 88.3 | 81.7 |
| Continuous | T5 | 16 | 91.7 | 90.0 | 91.7 | 66.7 | 81.7 | 84.3 |
| Dispersed | T4 | 5 | 88.3 | 70.0 | 63.3 | 68.3 | 73.3 | 72.7 |
| Dispersed | T4 | 10 | 88.3 | 93.3 | 95.0 | 85.0 | 90.0 | 90.3 |
| Dispersed | T4 | 14 | 75.0 | 81.7 | 78.3 | 86.7 | 88.3 | 82.0 |
| Dispersed | T5 | 2 | 75.0 | 71.7 | 51.7 | 80.0 | 68.3 | 69.3 |
| Dispersed | T5 | 4 | 51.7 | 53.3 | 36.7 | 41.7 | 1.7 | 37.0 |
| Dispersed | T5 | 6 | 83.3 | 88.3 | 93.3 | 88.3 | 91.7 | 89.0 |
| Dispersed | T5 | 10 | 91.7 | 96.7 | 95.0 | 93.3 | 90.0 | 93.3 |
| Dispersed | T5 | 12 | 86.7 | 75.0 | 93.3 | 73.3 | 63.3 | 78.3 |
| Dispersed | T5 | 15 | 90.0 | 96.7 | 91.7 | 86.7 | 88.3 | 90.7 |
|  |  |  |  |  |  |  |  |  |
| Broken cuff | T1 | 2 | 48.3 | 66.7 | 65.0 | 53.3 | 21.7 | 51.0 |
| Broken cuff | T1 | 7 | 86.7 | 86.7 | 86.7 | 68.3 | 61.7 | 78.0 |
| Broken cuff | T1 | 10 | 85.0 | 66.7 | 70.0 | 75.0 | 63.3 | 72.0 |
| Broken cuff | T4 | 7 | 90.0 | 78.3 | 85.0 | 88.3 | 86.7 | 85.7 |
| Broken cuff | T4 | 8 | 73.3 | 85.0 | 78.3 | 71.7 | 85.0 | 78.7 |
| Broken cuff | T4 | 13 | 88.3 | 91.7 | 88.3 | 78.3 | 85.0 | 86.3 |
| Excluded | T3 | 1 | 0.0 | 0.0 | 0.0 | 0.0 | 0.0 | 0.0 |
| Excluded | T3 | 7 | 0.0 | 0.0 | 0.0 | 0.0 | 0.0 | 0.0 |

**Extinction day 4**

|  | Cohort | Rat | CS1 | CS2 | CS3 | CS4 | CS5 | AVG |
| --- | --- | --- | --- | --- | --- | --- | --- | --- |
|  |  |  |  |  |  |  |  |  |
| SHAM | T1 | 5 | 56.7 | 65.0 | 61.7 | 65.0 | 68.3 | 63.3 |
| SHAM | T1 | 9 | 25.0 | 15.0 | 21.7 | 11.7 | 18.3 | 18.3 |
| SHAM | T1 | 14 | 83.3 | 88.3 | 71.7 | 65.0 | 80.0 | 77.7 |
| SHAM | T3 | 2 | 68.3 | 85.0 | 85.0 | 86.7 | 86.7 | 82.3 |
| SHAM | T3 | 8 | 58.3 | 40.0 | 78.3 | 53.3 | 71.7 | 60.3 |
| SHAM | T3 | 11 | 55.0 | 35.0 | 45.0 | 36.7 | 46.7 | 43.7 |
| SHAM | T3 | 14 | 70.0 | 83.3 | 90.0 | 50.0 | 55.0 | 69.7 |
| SHAM | T4 | 1 | 53.3 | 53.3 | 45.0 | 51.7 | 58.3 | 52.3 |
| SHAM | T4 | 6 | 83.3 | 90.0 | 81.7 | 68.3 | 66.7 | 78.0 |
| SHAM | T5 | 1 | 90.0 | 88.3 | 93.3 | 91.7 | 93.3 | 91.3 |
| SHAM | T5 | 7 | 81.7 | 91.7 | 75.0 | 13.3 | 83.3 | 69.0 |
| During | T1 | 1 | 41.7 | 43.3 | 46.7 | 20.0 | 38.3 | 38.0 |
| During | T1 | 3 | 75.0 | 66.7 | 48.3 | 63.3 | 88.3 | 68.3 |
| During | T1 | 11 | 26.7 | 35.0 | 16.7 | 21.7 | 21.7 | 24.3 |
| During | T1 | 13 | 41.7 | 43.3 | 23.3 | 15.0 | 5.0 | 25.7 |
| During | T1 | 16 | 28.3 | 35.0 | 20.0 | 33.3 | 25.0 | 28.3 |
| During | T3 | 4 | 63.3 | 58.3 | 55.0 | 51.7 | 48.3 | 55.3 |
| During | T3 | 6 | 41.7 | 55.0 | 38.3 | 25.0 | 38.3 | 39.7 |
| During | T3 | 13 | 51.7 | 75.0 | 85.0 | 73.3 | 88.3 | 74.7 |
| During | T4 | 15 | 51.7 | 60.0 | 53.3 | 45.0 | 31.7 | 48.3 |
| During | T5 | 5 | 41.7 | 55.0 | 31.7 | 51.7 | 25.0 | 41.0 |
| Between | T1 | 4 | 63.3 | 71.7 | 65.0 | 63.3 | 60.0 | 64.7 |
| Between | T1 | 6 | 81.7 | 71.7 | 71.7 | 71.7 | 66.7 | 72.7 |
| Between | T1 | 8 | 86.7 | 86.7 | 90.0 | 83.3 | 75.0 | 84.3 |
| Between | T1 | 12 | 80.0 | 81.7 | 80.0 | 86.7 | 83.3 | 82.3 |
| Between | T1 | 15 | 88.3 | 75.0 | 45.0 | 90.0 | 73.3 | 74.3 |
| Between | T3 | 3 | 56.7 | 25.0 | 50.0 | 66.7 | 55.0 | 50.7 |
| Between | T3 | 9 | 56.7 | 25.0 | 21.7 | 15.0 | 11.7 | 26.0 |
| Between | T3 | 15 | 48.3 | 63.3 | 70.0 | 61.7 | 41.7 | 57.0 |
| Between | T4 | 2 | 53.3 | 65.0 | 51.7 | 43.3 | 45.0 | 51.7 |
| Between | T4 | 11 | 68.3 | 90.0 | 38.3 | 81.7 | 85.0 | 72.7 |
| Between | T5 | 13 | 76.7 | 85.0 | 78.3 | 56.7 | 58.3 | 71.0 |
| Continuous | T3 | 5 | 68.3 | 66.7 | 46.7 | 51.7 | 50.0 | 56.7 |
| Continuous | T3 | 10 | 58.3 | 80.0 | 63.3 | 63.3 | 78.3 | 68.7 |
| Continuous | T3 | 12 | 5.0 | 18.3 | 1.7 | 0.0 | 11.7 | 7.3 |
| Continuous | T3 | 16 | 3.3 | 0.0 | 28.3 | 46.7 | 35.0 | 22.7 |
| Continuous | T4 | 4 | 43.3 | 43.3 | 36.7 | 36.7 | 33.3 | 38.7 |
| Continuous | T4 | 9 | 60.0 | 68.3 | 66.7 | 68.3 | 68.3 | 66.3 |
| Continuous | T4 | 12 | 50.0 | 45.0 | 53.3 | 38.3 | 56.7 | 48.7 |
| Continuous | T4 | 16 | 46.7 | 50.0 | 26.7 | 13.3 | 20.0 | 31.3 |
| Continuous | T5 | 8 | 90.0 | 66.7 | 60.0 | 28.3 | 26.7 | 54.3 |
| Continuous | T5 | 11 | 78.3 | 65.0 | 63.3 | 55.0 | 25.0 | 57.3 |
| Continuous | T5 | 16 | 70.0 | 53.3 | 38.3 | 63.3 | 61.7 | 57.3 |
| Dispersed | T4 | 5 | 71.7 | 58.3 | 25.0 | 58.3 | 38.3 | 50.3 |
| Dispersed | T4 | 10 | 73.3 | 68.3 | 65.0 | 33.3 | 53.3 | 58.7 |
| Dispersed | T4 | 14 | 53.3 | 51.7 | 63.3 | 20.0 | 30.0 | 43.7 |
| Dispersed | T5 | 2 | 71.7 | 86.7 | 90.0 | 78.3 | 60.0 | 77.3 |
| Dispersed | T5 | 4 | 16.7 | 45.0 | 65.0 | 26.7 | 21.7 | 35.0 |
| Dispersed | T5 | 6 | 86.7 | 86.7 | 80.0 | 83.3 | 65.0 | 80.3 |
| Dispersed | T5 | 10 | 91.7 | 93.3 | 96.7 | 91.7 | 96.7 | 94.0 |
| Dispersed | T5 | 12 | 68.3 | 78.3 | 78.3 | 48.3 | 46.7 | 64.0 |
| Dispersed | T5 | 15 | 90.0 | 75.0 | 80.0 | 76.7 | 90.0 | 82.3 |
|  |  |  |  |  |  |  |  |  |
| Broken cuff | T1 | 2 | 55.0 | 83.3 | 56.7 | 51.7 | 46.7 | 58.7 |
| Broken cuff | T1 | 7 | 75.0 | 65.0 | 60.0 | 68.3 | 85.0 | 70.7 |
| Broken cuff | T1 | 10 | 86.7 | 75.0 | 70.0 | 76.7 | 68.3 | 75.3 |
| Broken cuff | T4 | 7 | 65.0 | 71.7 | 78.3 | 65.0 | 75.0 | 71.0 |
| Broken cuff | T4 | 8 | 76.7 | 60.0 | 75.0 | 60.0 | 60.0 | 66.3 |
| Broken cuff | T4 | 13 | 56.7 | 50.0 | 60.0 | 68.3 | 56.7 | 58.3 |
| Excluded | T3 | 1 | 0.0 | 0.0 | 0.0 | 0.0 | 0.0 | 0.0 |
| Excluded | T3 | 7 | 0.0 | 0.0 | 0.0 | 0.0 | 0.0 | 0.0 |

**Extinction day 5**

|  | Cohort | Rat | CS1 | CS2 | CS3 | CS4 | CS5 | AVG |
| --- | --- | --- | --- | --- | --- | --- | --- | --- |
|  |  |  |  |  |  |  |  |  |
| SHAM | T1 | 5 | 83.3 | 83.3 | 61.7 | 75.0 | 63.3 | 73.3 |
| SHAM | T1 | 9 | 41.7 | 40.0 | 8.3 | 16.7 | 16.7 | 24.7 |
| SHAM | T1 | 14 | 70.0 | 60.0 | 48.3 | 80.0 | 75.0 | 66.7 |
| SHAM | T3 | 2 | 78.3 | 11.7 | 56.7 | 41.7 | 60.0 | 49.7 |
| SHAM | T3 | 8 | 55.0 | 61.7 | 48.3 | 83.3 | 55.0 | 60.7 |
| SHAM | T3 | 11 | 45.0 | 61.7 | 61.7 | 51.7 | 43.3 | 52.7 |
| SHAM | T3 | 14 | 66.7 | 70.0 | 46.7 | 43.3 | 36.7 | 52.7 |
| SHAM | T4 | 1 | 60.0 | 68.3 | 58.3 | 40.0 | 63.3 | 58.0 |
| SHAM | T4 | 6 | 83.3 | 81.7 | 66.7 | 70.0 | 73.3 | 75.0 |
| SHAM | T5 | 1 | 81.7 | 81.7 | 70.0 | 78.3 | 70.0 | 76.3 |
| SHAM | T5 | 7 | 71.7 | 76.7 | 56.7 | 56.7 | 30.0 | 58.3 |
| During | T1 | 1 | 28.3 | 11.7 | 20.0 | 33.3 | 6.7 | 20.0 |
| During | T1 | 3 | 58.3 | 48.3 | 43.3 | 83.3 | 86.7 | 64.0 |
| During | T1 | 11 | 13.3 | 25.0 | 10.0 | 5.0 | 1.7 | 11.0 |
| During | T1 | 13 | 28.3 | 30.0 | 18.3 | 23.3 | 11.7 | 22.3 |
| During | T1 | 16 | 16.7 | 20.0 | 15.0 | 21.7 | 10.0 | 16.7 |
| During | T3 | 4 | 53.3 | 51.7 | 48.3 | 41.7 | 50.0 | 49.0 |
| During | T3 | 6 | 41.7 | 16.7 | 30.0 | 23.3 | 18.3 | 26.0 |
| During | T3 | 13 | 26.7 | 31.7 | 33.3 | 15.0 | 26.7 | 26.7 |
| During | T4 | 15 | 13.3 | 36.7 | 26.7 | 0.0 | 15.0 | 18.3 |
| During | T5 | 5 | 30.0 | 40.0 | 20.0 | 25.0 | 25.0 | 28.0 |
| Between | T1 | 4 | 40.0 | 41.7 | 38.3 | 50.0 | 48.3 | 43.7 |
| Between | T1 | 6 | 83.3 | 66.7 | 85.0 | 86.7 | 81.7 | 80.7 |
| Between | T1 | 8 | 90.0 | 86.7 | 90.0 | 61.7 | 86.7 | 83.0 |
| Between | T1 | 12 | 53.3 | 41.7 | 46.7 | 38.3 | 36.7 | 43.3 |
| Between | T1 | 15 | 88.3 | 23.3 | 50.0 | 80.0 | 61.7 | 60.7 |
| Between | T3 | 3 | 48.3 | 23.3 | 38.3 | 43.3 | 43.3 | 39.3 |
| Between | T3 | 9 | 43.3 | 35.0 | 21.7 | 23.3 | 16.7 | 28.0 |
| Between | T3 | 15 | 51.7 | 43.3 | 38.3 | 18.3 | 43.3 | 39.0 |
| Between | T4 | 2 | 55.0 | 75.0 | 56.7 | 53.3 | 60.0 | 60.0 |
| Between | T4 | 11 | 65.0 | 65.0 | 61.7 | 50.0 | 68.3 | 62.0 |
| Between | T5 | 13 | 48.3 | 46.7 | 33.3 | 45.0 | 66.7 | 48.0 |
| Continuous | T3 | 5 | 58.3 | 56.7 | 58.3 | 80.0 | 48.3 | 60.3 |
| Continuous | T3 | 10 | 61.7 | 40.0 | 30.0 | 20.0 | 11.7 | 32.7 |
| Continuous | T3 | 12 | 15.0 | 26.7 | 23.3 | 21.7 | 43.3 | 26.0 |
| Continuous | T3 | 16 | 16.7 | 25.0 | 31.7 | 25.0 | 43.3 | 28.3 |
| Continuous | T4 | 4 | 73.3 | 33.3 | 70.0 | 20.0 | 8.3 | 41.0 |
| Continuous | T4 | 9 | 76.7 | 61.7 | 68.3 | 46.7 | 53.3 | 61.3 |
| Continuous | T4 | 12 | 53.3 | 55.0 | 53.3 | 46.7 | 43.3 | 50.3 |
| Continuous | T4 | 16 | 40.0 | 25.0 | 26.7 | 13.3 | 48.3 | 30.7 |
| Continuous | T5 | 8 | 38.3 | 23.3 | 43.3 | 26.7 | 15.0 | 29.3 |
| Continuous | T5 | 11 | 43.3 | 35.0 | 33.3 | 41.7 | 16.7 | 34.0 |
| Continuous | T5 | 16 | 30.0 | 40.0 | 30.0 | 41.7 | 26.7 | 33.7 |
| Dispersed | T4 | 5 | 56.7 | 18.3 | 61.7 | 56.7 | 51.7 | 49.0 |
| Dispersed | T4 | 10 | 51.7 | 48.3 | 46.7 | 55.0 | 60.0 | 52.3 |
| Dispersed | T4 | 14 | 71.7 | 75.0 | 76.7 | 66.7 | 40.0 | 66.0 |
| Dispersed | T5 | 2 | 88.3 | 75.0 | 41.7 | 43.3 | 63.3 | 62.3 |
| Dispersed | T5 | 4 | 26.7 | 16.7 | 20.0 | 13.3 | 16.7 | 18.7 |
| Dispersed | T5 | 6 | 60.0 | 53.3 | 58.3 | 68.3 | 56.7 | 59.3 |
| Dispersed | T5 | 10 | 91.7 | 91.7 | 100.0 | 95.0 | 88.3 | 93.3 |
| Dispersed | T5 | 12 | 53.3 | 65.0 | 60.0 | 48.3 | 46.7 | 54.7 |
| Dispersed | T5 | 15 | 83.3 | 80.0 | 66.7 | 55.0 | 76.7 | 72.3 |
|  |  |  |  |  |  |  |  |  |
| Broken cuff | T1 | 2 | 71.7 | 70.0 | 61.7 | 23.3 | 28.3 | 51.0 |
| Broken cuff | T1 | 7 | 85.0 | 48.3 | 70.0 | 71.7 | 68.3 | 68.7 |
| Broken cuff | T1 | 10 | 53.3 | 76.7 | 38.3 | 28.3 | 41.7 | 47.7 |
| Broken cuff | T4 | 7 | 78.3 | 70.0 | 68.3 | 71.7 | 91.7 | 76.0 |
| Broken cuff | T4 | 8 | 65.0 | 53.3 | 53.3 | 83.3 | 68.3 | 64.7 |
| Broken cuff | T4 | 13 | 78.3 | 45.0 | 68.3 | 45.0 | 48.3 | 57.0 |
| Excluded | T3 | 1 | 0.0 | 0.0 | 0.0 | 0.0 | 0.0 | 0.0 |
| Excluded | T3 | 7 | 0.0 | 0.0 | 0.0 | 0.0 | 0.0 | 0.0 |

**Extinction all days AVG**

|  | Cohort | Rat | AVG1 | AVG2 | AVG3 | AVG4 | AVG5 |
| --- | --- | --- | --- | --- | --- | --- | --- |
|  |  |  |  |  |  |  |  |
| SHAM | T1 | 5 | 75.7 | 59.7 | 64.7 | 63.3 | 73.3 |
| SHAM | T1 | 9 | 42.7 | 18.0 | 25.0 | 18.3 | 24.7 |
| SHAM | T1 | 14 | 79.3 | 68.3 | 76.3 | 77.7 | 66.7 |
| SHAM | T3 | 2 | 65.7 | 75.0 | 69.0 | 82.3 | 49.7 |
| SHAM | T3 | 8 | 50.7 | 84.0 | 44.3 | 60.3 | 60.7 |
| SHAM | T3 | 11 | 54.7 | 62.7 | 80.0 | 43.7 | 52.7 |
| SHAM | T3 | 14 | 58.0 | 85.3 | 72.7 | 69.7 | 52.7 |
| SHAM | T4 | 1 | 79.0 | 73.3 | 77.0 | 52.3 | 58.0 |
| SHAM | T4 | 6 | 84.3 | 75.3 | 85.7 | 78.0 | 75.0 |
| SHAM | T5 | 1 | 74.3 | 88.7 | 83.7 | 91.3 | 76.3 |
| SHAM | T5 | 7 | 73.7 | 87.3 | 85.7 | 69.0 | 58.3 |
| During | T1 | 1 | 79.0 | 65.3 | 69.0 | 38.0 | 20.0 |
| During | T1 | 3 | 71.3 | 39.3 | 61.3 | 68.3 | 64.0 |
| During | T1 | 11 | 28.3 | 19.0 | 24.7 | 24.3 | 11.0 |
| During | T1 | 13 | 51.3 | 19.0 | 29.3 | 25.7 | 22.3 |
| During | T1 | 16 | 85.3 | 53.7 | 39.0 | 28.3 | 16.7 |
| During | T3 | 4 | 74.3 | 74.0 | 48.7 | 55.3 | 49.0 |
| During | T3 | 6 | 73.0 | 60.0 | 35.0 | 39.7 | 26.0 |
| During | T3 | 13 | 52.7 | 55.3 | 28.3 | 74.7 | 26.7 |
| During | T4 | 15 | 69.7 | 75.3 | 77.3 | 48.3 | 18.3 |
| During | T5 | 5 | 71.3 | 83.3 | 79.0 | 41.0 | 28.0 |
| Between | T1 | 4 | 69.7 | 51.0 | 35.7 | 64.7 | 43.7 |
| Between | T1 | 6 | 68.3 | 61.0 | 51.3 | 72.7 | 80.7 |
| Between | T1 | 8 | 77.3 | 74.7 | 70.0 | 84.3 | 83.0 |
| Between | T1 | 12 | 78.0 | 50.0 | 59.3 | 82.3 | 43.3 |
| Between | T1 | 15 | 70.0 | 28.7 | 48.0 | 74.3 | 60.7 |
| Between | T3 | 3 | 47.7 | 55.0 | 23.0 | 50.7 | 39.3 |
| Between | T3 | 9 | 58.0 | 57.0 | 42.0 | 26.0 | 28.0 |
| Between | T3 | 15 | 74.3 | 74.7 | 53.3 | 57.0 | 39.0 |
| Between | T4 | 2 | 69.3 | 65.7 | 75.7 | 51.7 | 60.0 |
| Between | T4 | 11 | 87.3 | 78.7 | 89.0 | 72.7 | 62.0 |
| Between | T5 | 13 | 73.7 | 80.3 | 66.3 | 71.0 | 48.0 |
| Continuous | T3 | 5 | 55.0 | 76.7 | 51.3 | 56.7 | 60.3 |
| Continuous | T3 | 10 | 79.0 | 73.0 | 59.3 | 68.7 | 32.7 |
| Continuous | T3 | 12 | 73.3 | 74.0 | 32.3 | 7.3 | 26.0 |
| Continuous | T3 | 16 | 49.0 | 62.3 | 24.3 | 22.7 | 28.3 |
| Continuous | T4 | 4 | 71.7 | 62.0 | 83.7 | 38.7 | 41.0 |
| Continuous | T4 | 9 | 81.7 | 72.3 | 82.0 | 66.3 | 61.3 |
| Continuous | T4 | 12 | 81.0 | 78.7 | 82.7 | 48.7 | 50.3 |
| Continuous | T4 | 16 | 79.3 | 85.0 | 49.3 | 31.3 | 30.7 |
| Continuous | T5 | 8 | 81.0 | 82.3 | 80.7 | 54.3 | 29.3 |
| Continuous | T5 | 11 | 77.7 | 89.3 | 81.7 | 57.3 | 34.0 |
| Continuous | T5 | 16 | 69.7 | 92.0 | 84.3 | 57.3 | 33.7 |
| Dispersed | T4 | 5 | 72.3 | 75.0 | 72.7 | 50.3 | 49.0 |
| Dispersed | T4 | 10 | 90.3 | 84.7 | 90.3 | 58.7 | 52.3 |
| Dispersed | T4 | 14 | 71.3 | 82.3 | 82.0 | 43.7 | 66.0 |
| Dispersed | T5 | 2 | 66.7 | 62.3 | 69.3 | 77.3 | 62.3 |
| Dispersed | T5 | 4 | 51.3 | 59.0 | 37.0 | 35.0 | 18.7 |
| Dispersed | T5 | 6 | 81.7 | 87.0 | 89.0 | 80.3 | 59.3 |
| Dispersed | T5 | 10 | 82.3 | 90.7 | 93.3 | 94.0 | 93.3 |
| Dispersed | T5 | 12 | 79.7 | 83.3 | 78.3 | 64.0 | 54.7 |
| Dispersed | T5 | 15 | 90.0 | 95.3 | 90.7 | 82.3 | 72.3 |
|  |  |  |  |  |  |  |  |
| Broken cuff | T1 | 2 | 61.0 | 58.7 | 51.0 | 58.7 | 51.0 |
| Broken cuff | T1 | 7 | 84.7 | 37.0 | 78.0 | 70.7 | 68.7 |
| Broken cuff | T1 | 10 | 49.3 | 62.0 | 72.0 | 75.3 | 47.7 |
| Broken cuff | T4 | 7 | 85.3 | 79.0 | 85.7 | 71.0 | 76.0 |
| Broken cuff | T4 | 8 | 79.0 | 76.0 | 78.7 | 66.3 | 64.7 |
| Broken cuff | T4 | 13 | 77.7 | 80.7 | 86.3 | 58.3 | 57.0 |
| Excluded | T3 | 1 | lost headcap | |  |  |  |
| Excluded | T3 | 7 | lost headcap | |  |  |  |

**Retention session**

|  | Cohort | Rat | CS1 | CS2 | CS3 | CS4 | CS5 | AVG |
| --- | --- | --- | --- | --- | --- | --- | --- | --- |
|  |  |  |  |  |  |  |  |  |
| SHAM | T1 | 5 | 83.3 | 86.7 | 66.7 | 43.3 | 36.7 | 63.3 |
| SHAM | T1 | 9 | 80.0 | 90.0 | 50.0 | 26.7 | 30.0 | 55.3 |
| SHAM | T1 | 14 | 56.7 | 60.0 | 60.0 | 63.3 | 6.7 | 49.3 |
| SHAM | T3 | 2 | 63.3 | 58.3 | 55.0 | 61.7 | 63.3 | 60.3 |
| SHAM | T3 | 8 | 16.7 | 16.7 | 20.0 | 26.7 | 31.7 | 22.3 |
| SHAM | T3 | 11 | 70.0 | 58.3 | 60.0 | 25.0 | 53.3 | 53.3 |
| SHAM | T3 | 14 | 80.0 | 38.3 | 81.7 | 76.7 | 81.7 | 71.7 |
| SHAM | T4 | 1 | 61.7 | 45.0 | 41.7 | 50.0 | 40.0 | 47.7 |
| SHAM | T4 | 6 | 83.3 | 78.3 | 66.7 | 15.0 | 71.7 | 63.0 |
| SHAM | T5 | 1 | 91.7 | 81.7 | 48.3 | 81.7 | 48.3 | 70.3 |
| SHAM | T5 | 7 | 96.7 | 78.3 | 76.7 | 70.0 | 78.3 | 80.0 |
| During | T1 | 1 | 20.0 | 60.0 | 10.0 | 13.3 | 6.7 | 22.0 |
| During | T1 | 3 | 6.7 | 30.0 | 0.0 | 0.0 | 6.7 | 8.7 |
| During | T1 | 11 | 6.7 | 0.0 | 10.0 | 0.0 | 0.0 | 3.3 |
| During | T1 | 13 | 66.7 | 40.0 | 56.7 | 26.7 | 43.3 | 46.7 |
| During | T1 | 16 | 33.3 | 16.7 | 6.7 | 0.0 | 0.0 | 11.3 |
| During | T3 | 4 | 55.0 | 30.0 | 51.7 | 45.0 | 63.3 | 49.0 |
| During | T3 | 6 | 13.3 | 1.7 | 8.3 | 13.3 | 31.7 | 13.7 |
| During | T3 | 13 | 66.7 | 63.3 | 73.3 | 23.3 | 40.0 | 53.3 |
| During | T4 | 15 | 48.3 | 28.3 | 28.3 | 58.3 | 13.3 | 35.3 |
| During | T5 | 5 | 68.3 | 76.7 | 66.7 | 50.0 | 76.7 | 67.7 |
| Between | T1 | 4 | 55.0 | 61.7 | 56.7 | 60.0 | 33.3 | 53.3 |
| Between | T1 | 6 | 60.0 | 66.7 | 36.7 | 86.7 | 70.0 | 64.0 |
| Between | T1 | 8 | 53.3 | 63.3 | 73.3 | 56.7 | 66.7 | 62.7 |
| Between | T1 | 12 | 50.0 | 63.3 | 33.3 | 33.3 | 36.7 | 43.3 |
| Between | T1 | 15 | 70.0 | 16.7 | 50.0 | 0.0 | 30.0 | 33.3 |
| Between | T3 | 3 | 43.3 | 41.7 | 48.3 | 30.0 | 35.0 | 39.7 |
| Between | T3 | 9 | 15.0 | 3.3 | 21.7 | 26.7 | 26.7 | 18.7 |
| Between | T3 | 15 | 33.3 | 51.7 | 63.3 | 23.3 | 20.0 | 38.3 |
| Between | T4 | 2 | 70.0 | 28.3 | 16.7 | 13.3 | 25.0 | 30.7 |
| Between | T4 | 11 | 91.7 | 90.0 | 86.7 | 85.0 | 73.3 | 85.3 |
| Between | T5 | 13 | 26.7 | 30.0 | 23.3 | 40.0 | 5.0 | 25.0 |
| Continuous | T3 | 5 | 51.7 | 38.3 | 31.7 | 46.7 | 20.0 | 37.7 |
| Continuous | T3 | 10 | 70.0 | 51.7 | 5.0 | 38.3 | 30.0 | 39.0 |
| Continuous | T3 | 12 | 10.0 | 1.7 | 6.7 | 33.3 | 3.3 | 11.0 |
| Continuous | T3 | 16 | 6.7 | 1.7 | 18.3 | 13.3 | 5.0 | 9.0 |
| Continuous | T4 | 4 | 33.3 | 43.3 | 5.0 | 25.0 | 6.7 | 22.7 |
| Continuous | T4 | 9 | 73.3 | 71.7 | 68.3 | 55.0 | 75.0 | 68.7 |
| Continuous | T4 | 12 | 65.0 | 51.7 | 60.0 | 50.0 | 40.0 | 53.3 |
| Continuous | T4 | 16 | 51.7 | 11.7 | 8.3 | 21.7 | 31.7 | 25.0 |
| Continuous | T5 | 8 | 15.0 | 1.7 | 1.7 | 11.7 | 1.7 | 6.3 |
| Continuous | T5 | 11 | 46.7 | 0.0 | 10.0 | 5.0 | 35.0 | 19.3 |
| Continuous | T5 | 16 | 70.0 | 73.3 | 25.0 | 16.7 | 68.3 | 50.7 |
| Dispersed | T4 | 5 | 81.7 | 80.0 | 83.3 | 70.0 | 51.7 | 73.3 |
| Dispersed | T4 | 10 | 65.0 | 71.7 | 40.0 | 18.3 | 65.0 | 52.0 |
| Dispersed | T4 | 14 | 81.7 | 58.3 | 56.7 | 51.7 | 80.0 | 65.7 |
| Dispersed | T5 | 2 | 76.7 | 20.0 | 70.0 | 40.0 | 56.7 | 52.7 |
| Dispersed | T5 | 4 | 11.7 | 5.0 | 6.7 | 0.0 | 40.0 | 12.7 |
| Dispersed | T5 | 6 | 73.3 | 88.3 | 86.7 | 66.7 | 65.0 | 76.0 |
| Dispersed | T5 | 10 | 91.7 | 91.7 | 83.3 | 90.0 | 91.7 | 89.7 |
| Dispersed | T5 | 12 | 73.3 | 76.7 | 58.3 | 40.0 | 40.0 | 57.7 |
| Dispersed | T5 | 15 | 88.3 | 60.0 | 73.3 | 30.0 | 38.3 | 58.0 |
|  |  |  |  |  |  |  |  |  |
| Broken cuff | T1 | 2 | 33.3 | 26.7 | 10.0 | 40.0 | 36.7 | 29.3 |
| Broken cuff | T1 | 7 | 66.7 | 33.3 | 6.7 | 16.7 | 50.0 | 34.7 |
| Broken cuff | T1 | 10 | 86.7 | 63.3 | 33.3 | 30.0 | 40.0 | 50.7 |
| Broken cuff | T4 | 7 | 85.0 | 88.3 | 91.7 | 88.3 | 90.0 | 88.7 |
| Broken cuff | T4 | 8 | 73.3 | 78.3 | 83.3 | 28.3 | 43.3 | 61.3 |
| Broken cuff | T4 | 13 | 91.7 | 91.7 | 63.3 | 85.0 | 76.7 | 81.7 |
| Excluded | T3 | 1 |  |  |  |  |  |  |
| Excluded | T3 | 7 |  |  |  |  |  |  |

**Improvement**

|  |  |  |  |  |  | % Improvement |
| --- | --- | --- | --- | --- | --- | --- |
|  |  |  | Day 1 | Day 5 | Retention | Day 1-Ret |
| SHAM | T1 | 5 | 75.7 | 73.3 | 63.3 | 16.3 |
| SHAM | T1 | 9 | 42.7 | 24.7 | 55.3 | -29.7 |
| SHAM | T1 | 14 | 79.3 | 66.7 | 49.3 | 37.8 |
| SHAM | T3 | 2 | 65.7 | 49.7 | 60.3 | 8.1 |
| SHAM | T3 | 8 | 50.7 | 60.7 | 22.3 | 55.9 |
| SHAM | T3 | 11 | 54.7 | 52.7 | 53.3 | 2.4 |
| SHAM | T3 | 14 | 58.0 | 52.7 | 71.7 | -23.6 |
| SHAM | T4 | 1 | 79.0 | 58.0 | 47.7 | 39.7 |
| SHAM | T4 | 6 | 84.3 | 75.0 | 63.0 | 25.3 |
| SHAM | T5 | 1 | 74.3 | 76.3 | 70.3 | 5.4 |
| SHAM | T5 | 7 | 73.7 | 58.3 | 80.0 | -8.6 |
|  |  |  |  |  |  | 11.7 |
|  |  |  |  |  |  |  |
|  |  |  |  |  |  |  |
|  |  |  | Day 1 | Day 5 | Retention | Day 1-Ret |
| During | T1 | 1 | 79.0 | 20.0 | 22.0 | 72.2 |
| During | T1 | 3 | 71.3 | 64.0 | 8.7 | 87.9 |
| During | T1 | 11 | 28.3 | 11.0 | 3.3 | 88.2 |
| During | T1 | 13 | 51.3 | 22.3 | 46.7 | 9.1 |
| During | T1 | 16 | 85.3 | 16.7 | 11.3 | 86.7 |
| During | T3 | 4 | 74.3 | 49.0 | 49.0 | 34.1 |
| During | T3 | 6 | 73.0 | 26.0 | 13.7 | 81.3 |
| During | T3 | 13 | 52.7 | 26.7 | 53.3 | -1.3 |
| During | T4 | 15 | 69.7 | 18.3 | 35.3 | 49.3 |
| During | T5 | 5 | 71.3 | 28.0 | 67.7 | 5.1 |
|  |  |  |  |  |  | 51.3 |
|  |  |  |  |  |  |  |
|  |  |  |  |  |  |  |
|  |  |  |  |  |  |  |
|  |  |  | Day 1 | Day 5 | Retention | Day 1-Ret |
| Between | T1 | 4 | 69.7 | 43.7 | 53.3 | 23.4 |
| Between | T1 | 6 | 68.3 | 80.7 | 64.0 | 6.3 |
| Between | T1 | 8 | 77.3 | 83.0 | 62.7 | 19.0 |
| Between | T1 | 12 | 78.0 | 43.3 | 43.3 | 44.4 |
| Between | T1 | 15 | 70.0 | 60.7 | 33.3 | 52.4 |
| Between | T3 | 3 | 47.7 | 39.3 | 39.7 | 16.8 |
| Between | T3 | 9 | 58.0 | 28.0 | 18.7 | 67.8 |
| Between | T3 | 15 | 74.3 | 39.0 | 38.3 | 48.4 |
| Between | T4 | 2 | 69.3 | 60.0 | 30.7 | 55.8 |
| Between | T4 | 11 | 87.3 | 62.0 | 85.3 | 2.3 |
| Between | T5 | 13 | 73.7 | 48.0 | 25.0 | 66.1 |
|  |  |  |  |  |  | 36.6 |
|  |  |  |  |  |  |  |
|  |  |  |  |  |  |  |
|  |  |  |  |  |  |  |
|  |  |  | Day 1 | Day 5 | Retention | Day 1-Ret |
| Dispersed | T4 | 5 | 72.3 | 49.0 | 73.3 | -1.4 |
| Dispersed | T4 | 10 | 90.3 | 52.3 | 52.0 | 42.4 |
| Dispersed | T4 | 14 | 71.3 | 66.0 | 65.7 | 7.9 |
| Dispersed | T5 | 2 | 66.7 | 62.3 | 52.7 | 21.0 |
| Dispersed | T5 | 4 | 51.3 | 18.7 | 12.7 | 75.3 |
| Dispersed | T5 | 6 | 81.7 | 59.3 | 76.0 | 6.9 |
| Dispersed | T5 | 10 | 82.3 | 93.3 | 89.7 | -8.9 |
| Dispersed | T5 | 12 | 79.7 | 54.7 | 57.7 | 27.6 |
| Dispersed | T5 | 15 | 90.0 | 72.3 | 58.0 | 35.6 |
|  |  |  |  |  |  | 22.9 |
|  |  |  |  |  |  |  |
|  |  |  |  |  |  |  |
|  |  |  | Day 1 | Day 5 | Retention | Day 1-Ret |
| Continuous | T3 | 5 | 55.0 | 60.3 | 37.7 | 31.5 |
| Continuous | T3 | 10 | 79.0 | 32.7 | 39.0 | 50.6 |
| Continuous | T3 | 12 | 73.3 | 26.0 | 11.0 | 85.0 |
| Continuous | T3 | 16 | 49.0 | 28.3 | 9.0 | 81.6 |
| Continuous | T4 | 4 | 71.7 | 41.0 | 22.7 | 68.4 |
| Continuous | T4 | 9 | 81.7 | 61.3 | 68.7 | 15.9 |
| Continuous | T4 | 12 | 81.0 | 50.3 | 53.3 | 34.2 |
| Continuous | T4 | 16 | 79.3 | 30.7 | 25.0 | 68.5 |
| Continuous | T5 | 8 | 81.0 | 29.3 | 6.3 | 92.2 |
| Continuous | T5 | 11 | 77.7 | 34.0 | 19.3 | 75.1 |
| Continuous | T5 | 16 | 69.7 | 33.7 | 50.7 | 27.3 |
|  |  |  |  |  |  | 57.3 |
|  |  |  |  |  |  |  |
